# A Comprehensive Instrumental Analysis Framework for Assessing the Dissolvability and Taste Properties of Plant Extract Instant Granules

**DOI:** 10.3390/foods15112000

**Published:** 2026-06-03

**Authors:** Xiao Ma, Zhaozhou Lin, Yidan Wang, Hui Jiang, Juntao Xie, Qifei Gu, Yifan Hu, Gan Luo, Bing Xu

**Affiliations:** 1 Department of Chinese Medicine Informatics, Beijing University of Chinese Medicine, Beijing 100029, China; 20230935203@bucm.edu.cn (X.M.); 20240935146@bucm.edu.cn (Y.W.); 20240935282@bucm.edu.cn (H.J.); 20240935221@bucm.edu.cn (J.X.); 20250941619@bucm.edu.cn (Q.G.); 20240935222@bucm.edu.cn (Y.H.); 2 Beijing Key Laboratory of Chinese Medicine Manufacturing Process Control and Quality Evaluation, Beijing University of Chinese Medicine, Beijing 100029, China; 3 Department of Drug Screening, Beijing Tongrentang Technology Development Co., Ltd., Beijing 100079, China; linzhaozhou@bucm.edu.cn

**Keywords:** instant granules, rehydration performance, electronic tongue, multi-criteria evaluation, analytic hierarchy process, quality assessment

## Abstract

Flavor profile and water dissolvability serve as core evaluation benchmarks for the quality of food and medicinal plant-derived instant granules. Currently, studies integrating flavor and dissolvability analysis to comprehensively characterize the overall performance of such granules remain scarce, and the existing literature lacks systematic comparative research on commercial products across multiple sources and batches. This study investigated 90 batches of four categories of plant extract instant granules and established a dynamic-static joint evaluation system coupled with multiple indicators and the Analytic Hierarchy Process (AHP). The three primary indicators were dissolving extent, dissolving rate, and taste, with equal weights assigned to each; the secondary indicators were classified and integrated based on the results of Principal Component Analysis (PCA) and correlation matrix. Quantitative analysis revealed that the comprehensive evaluation scores of all 90 batches of samples fluctuated between 0.5406 and 0.9503, and obvious disparities existed among different granule varieties. This multi-index evaluation framework effectively avoids the subjective bias inherent in conventional evaluation approaches, and lays a solid scientific foundation for quality supervision, formula optimization research and development, as well as market popularization of plant-based instant granules.

## 1. Introduction

Shifts in modern lifestyles have driven surging demand for instant powders/granules, fueled by consumers’ expectations for convenience, nutrition, and advanced processing [1,2,3]. Produced by dehydrating raw materials, these products offer easy reconstitution, transport, and extended shelf life [4]. Plant extract granules, used as functional foods (coffee, tea [5,6,7]) and therapeutic agents (Chinese herbal, Kampo granules [8,9,10]), have seen steady global growth: China’s 2024 herbal formula granule market reached $9.25 billion (over 300 national standards [11]); Japan’s 2024 Kampo granule market hit 1.45 billion (2.0% of prescription sales [12]); and the 2025 global ready-to-drink tea/coffee market reached $52.84 billion, led by Asia-Pacific and fast-growing North America [13,14].

Flavor and dissolvability are two core attributes tied to consumer preference. Flavor studies support sensory optimization, quality control, and new formulation development [15]. Beyond subjective panel evaluations, objective techniques like NIR spectroscopy and UPLC-MS characterize flavor-related chemical fingerprints [16,17,18,19]. For example, UPLC-MS analysis linked chlorogenic acid levels in black instant coffee to color, flavor, and aroma [20]. Electronic tongues, simulating human taste perception, enable precise quantitative analysis of bitterness in herbs (e.g., chrysanthemum, licorice [21,22]). Integrating sensory technologies with chemometrics/AI further predicts consumer preferences [23,24,25]. Dissolvability (rate and extent) impacts perceived convenience and mouthfeel uniformity. Rapid dissolution ensures quick preparation and consistent flavor/texture. The process involves wetting, sinking, dispersion, and solubilization [26,27,28,29]. While milk powder rehydration is well-studied (via dispersibility indexes, light scattering, etc. [30,31,32]), granulation has a “double-edged sword” effect: it creates pores to aid dissolution but reduces surface area, potentially slowing it down.

Despite notable advancements in the evaluation of plant-derived instant products, some critical research gaps remain unaddressed. Firstly, there is a striking lack of integrated analytical frameworks that simultaneously characterize both flavor profiles and dissolvability performance for plant extract granules. Then, the public literature on granule rehydration behavior remains extremely limited, as most manufacturers treat related evaluation technologies and data as proprietary intellectual property to maintain competitive advantages. To address these, this study analyzes diverse plant-derived granules from multiple sources/batches. We innovatively developed a dynamic rehydration test system to simulate dissolution, paired with an electronic tongue and a turbidity sensor for static flavor analysis. Using the analytic hierarchy process on multidimensional data, we establish a comprehensive quantitative evaluation framework. This overcomes traditional method subjectivity, providing a scientific basis for quality control, targeted R&D, and market acceptance improvement.

## 2. Materials and Methods

### 2.1. Materials

This study utilized a total of 90 batches of plant extract granules, including 50 batches of multi-plant extract granules and 40 batches of single-plant extract granules. Among the multi-plant extract granules, 30 batches are Chinese herbal granules and 20 batches are Kampo granules. The single-plant extract granules included 30 batches of self-made single-extract granules and 10 batches of coffee granules. The details of these 90 batches of granules are listed in Tables S1–S4. Notably, among the Chinese herbal granules, Sanhan Huashi granules, Kunxinning granules, and Huashi Baidu granules were approved within the last five years. A total of 20 batches of Kampo granules were bought from Tsumura & Co., Ltd., Tokyo, Japan; Sanwa Yakuhin Co., Ltd., Tokyo, Japan; and Kracie Inc.,Tokyo, Japan, respectively. Additionally, 10 batches of freeze-dried coffee granules were bought from the market. Finally, 30 batches of self-made single-extract granules were prepared in-house by the research team. The raw extract powders were provided by Tcmages Co., Ltd., Beijing, China, and were produced at pilot scale through standardized processes comprising water extraction, solid–liquid separation, concentration, and spray drying. It should be highlighted that the inclusion of 90 batches of samples spanning four highly heterogeneous categories—namely complex multi-herb formulas (CHG and KG), single-plant extracts (SSEG), and non-herbal, lipid-containing matrices (CG)—represents a deliberate methodological choice for unified modeling. The central objective of executing a direct unified analysis across these diverse products, rather than treating them separately, is to thoroughly evaluate the universal applicability and mathematical robustness of the proposed joint framework. By testing the system under contrasting physicochemical baselines (e.g., sticky herbal polysaccharides vs. roasted coffee fractions vs. water-insoluble starch excipients), we intended to establish a broad-spectrum tool capable of abstracting core rehydration and gustatory rules, thereby ensuring the model is practical and meaningful.

### 2.2. On-Line Particle Size Distribution Test

**Figure 1:**
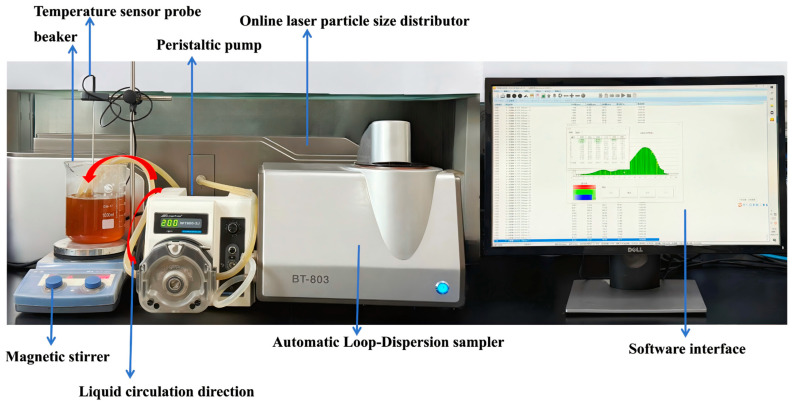
Schematic diagram of online particle size distribution test.

A 1000 mL beaker was placed on an RCT B S025 thermostatic magnetic stirrer (IKA, Staufen, Germany). A total of 900 mL of hot water was added, and the stirring speed was set to 150 rpm to initiate rotor agitation. The WT600-2J peristaltic pump (Baoding Longer Pump LTD., Baoding, China) was then activated. One end of the tubing was immersed in the beaker, while the other end was connected to the laser particle size analyzer (Dandong Bettersize Instruments LTD., Dandong, China). When the temperature of the stirred solution reached 75 °C, the measurement software of the laser particle size analyzer (Bettersize Laser Particle Size Analyzer Analysis System V8.10) was launched, and the testing method was configured. The experimental setup is shown in Figure 1. Upon starting the test and completing the background measurement, an appropriate amount of granules was immediately introduced. Particle size data were recorded in triplicate continuously from the beginning of the test until 600 s. *Span* values, a parameter reflecting particle size uniformity, was calculated in accordance with Formula (1). Based on the dynamic change curve of *D*_50_ values over time, the half-life of granules dissolving (*T*_50_), is defined as the time elapsed from the initial moment until the *D*_50_ values (i.e., the median particle size) of the granule size decreases to half its initial value.

(1) $$Span\;=\;\frac{D_{90}\;-\;D_{10}}{D_{50}}$$

### 2.3. In-Line Turbidity Test

**Figure 2:**
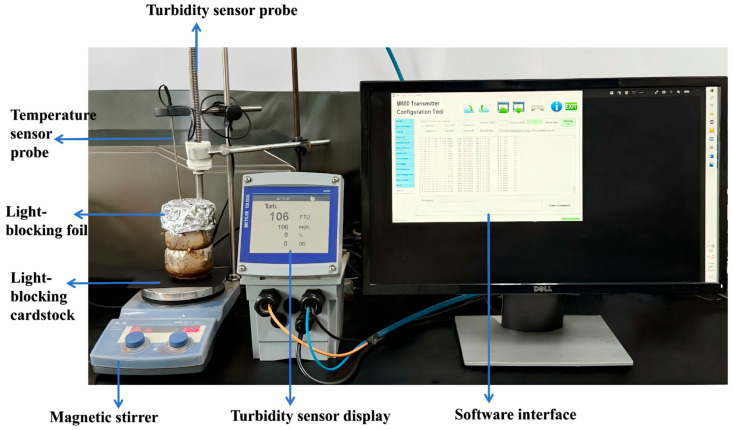
Schematic diagram of in-line turbidity testing.

A 250 mL tall beaker, protected from light, was filled with 200 mL of water and placed on an RCT B S025 thermostatic magnetic stirrer (IKA, Staufen, Germany). The Inpro8200/S/Epoxy/120 turbidity sensor (Mettler Toledo Instruments Co., Ltd., Greifensee, Switzerland) was inserted vertically into the beaker, positioned 1.5 cm from the rim. The tip of the sensor probe was adjusted to approximately 0.5 cm below the liquid surface and maintained at least 7 cm from the bottom of the beaker. Following sensor placement, a B-type rotor was introduced into the beaker. Stirring was initiated at 400 rpm, and the magnetic stirrer temperature was set to 75 °C. Once the water temperature stabilized, the M800TCT software (V1.8.0.0) was launched. The sample addition procedure was activated, after which approximately 10 g of granular sample was accurately weighed and gradually introduced into the beaker, and this process was performed in triplicate. Upon addition, the beaker was covered with aluminum foil to shield it from light. The experimental setup is shown in Figure 2. Prior to testing, the sensor was calibrated using a series of formalin-based standard turbidity solutions at 0, 500, 1000, 2000, and 4000 FTU. The time-to-turbidity equilibrium state (*T*_e_) is determined by analyzing the first derivative of the turbidity curve over time. At the equilibrium state, the first derivative value should approach zero or exhibit random fluctuations near zero.

### 2.4. Dissolution Test

The dissolution test was performed using an RC806D dissolution apparatus (TianDa TianFa Technology Co., Ltd., Tianjin, China). Initially, 900 mL of deionized water was introduced into a 1000 mL dissolution vessel, and the temperature was set to 37 °C with a stirring speed of 100 rpm. Approximately 1.5 g of sample granules were coarsely ground and passed through a 100-mesh sieve to obtain the fine powder. About 1 g of this fine powder was accurately weighed and added to the dissolution vessel to achieve complete dissolution. At the 60 min time point, 10 mL aliquots were withdrawn in triplicate. Each aliquot was immediately filtered through a 0.45 μm microporous membrane filter, and the resulting filtrates were pooled as the reference solution. Concurrently, 1 g of the original sample Granule (unground) was added to another dissolution vessel containing 900 mL of deionized water under the same temperature and stirring conditions. After each withdrawal, an equal volume of prewarmed deionized water (37 °C) was immediately replenished. Each sample was promptly filtered through a 0.45 μm microporous membrane to prevent continued dissolution of particulates post-sampling. The collected filtrates constituted the sample solutions. Both the reference solution and the sample solutions were appropriately diluted to ensure absorbance values fell within the range of 0.2 to 0.8. The diluted solutions were analyzed using a TU-1900 UV-Vis spectrophotometer (Beijing Persee General Instrument Co., Ltd., Beijing, China) across a wavelength range from 190 to 380 nm. Absorbance values at the maximum absorption wavelength were recorded at 0.5, 1, 1.5, 2, 3, 5, and 60 min. The cumulative dissolution percentage at each time point was calculated according to Formula (2). All tests were performed in triplicate.

Cumulative dissolution

(2) $$\left(Q_{j}\right)\;=\frac{{\;m_{i}\times A}_{i}\times W_{s}}{m_{s}\times A_{s}\times W_{i}}\;\times \;100\%$$

In the above formula, *A_i_* represents the absorbance of the sample solution, *m_i_* denotes the dilution factor of the sample solution, and *W_i_* indicates the mass of the test sample; *A*_s_ signifies the absorbance of the control solution at the same wavelength as the sample, *m*_s_ indicates the dilution factor of the control solution, and *W*_s_ denotes the mass of the control sample.

### 2.5. Approximate Solubility Test

Approximately 0.5 g of granules was accurately weighed and transferred into a 50 mL centrifuge tube containing 10 mL of boiling deionized water. The mixture was stirred for 5 min and followed by centrifugation. Centrifugation was performed using a DT5-2B centrifuge (Hebei Yizhong Medical Devices Co., Ltd., Shijiazhuang, China) at 1500 *g* for 15 min at 25 °C. Following centrifugation, the supernatant was decanted into an evaporating dish that had been previously dried to constant weight. The sample was evaporated to dryness on a HH-6 water bath (Shanghai Lichen Bangxi Instrument Technology Co., Ltd., Shanghai, China), after which the evaporating dish was transferred to a 105 °C electric forced-air drying oven until constant weight was achieved. The dish containing the dried residue was precisely weighed, and the proportion of soluble solids termed as approximate solubility was calculated according to Formula (3).

(3) $$\mathrm{Approximate}\;\mathrm{solubility}=\;\frac{\mathrm{Weight}\;\mathrm{of}\;\mathrm{soluble}\;\mathrm{solids}}{\mathrm{Total}\;\mathrm{weight}\;\mathrm{of}\;\mathrm{granules}}\;\times \;100\%$$

### 2.6. Taste Sensing System/Electronic Tongue

“Taste” specifically refers to the taste perception results obtained by electronic tongue simulation of human gustatory sensation. Taste characteristics of the granule solutions from 90 batches were analyzed using an SA-402B taste analyzer (Intelligent Sensor Technology, Inc., Kanagawa, Japan). The electronic tongue system was equipped with five taste sensors (Table 1) and two Ag/AgCl reference electrodes. Both the reference and sensor electrodes were filled with an internal solution consisting of 3.33 M KCl and saturated AgCl. Prior to measurement, the detection unit was immersed in a reference solution (30 mM KCl, 0.3 mM tartaric acid) to acquire a baseline potential (*V*_r_). This reference solution simulates human saliva and provides a neutral reference for taste intensity. The sensor electrodes were then immersed in the sample solution to record the sample potential (*V*_s_). The potential difference (*V*_s_ − *V*_r_) was defined as the response value, which was directly correlated with the intensity of the corresponding taste attribute.

An accurately weighed 2 g portion of granules was transferred into an Erlenmeyer flask, dissolved, and diluted to 100 mL with distilled water. The solution was magnetically stirred at 75 °C and 400 rpm for 10 min, followed by ultrasonic extraction at 30 °C for 30 min. After cooling to room temperature, the mixture was shaken thoroughly, filtered, and the filtrate was collected. Approximately 35 mL of the filtrate was transferred into a dedicated beaker for electronic tongue analysis at ambient temperature. After each measurement, the sensor electrode membranes were rinsed with a cleaning solution to remove adsorbed residues from the previous sample. Each sample was measured four times in succession, and data from the 2nd to 4th determinations were used for subsequent data analysis to minimize experimental variation and improve result reliability.

### 2.7. Analytic Hierarchy Process

The Analytic Hierarchy Process was used to establish a comprehensive evaluation method [33]. The Analytic Hierarchy Process (AHP) is a qualitative and quantitative analytical method that constructs an ordered hierarchical structure based on evaluation indicators for assigning scores. Evaluation indicators are paired to form a judgment matrix, with pairwise comparisons of each indicator’s importance relative to its counterparts at the same level within each layer. Just as stated in the previous reference [33], the procedure of AHP is briefly as follows. Firstly, the overall evaluation goal is continuously decomposed to derive evaluation goals at different levels. Then, each level’s evaluation goal is organically displayed in the diagram to form the goal tree. Next, weight coefficients are calculated. Within each level, evaluation targets are assigned specific weights based on their relative contribution to the value of the preceding level’s target. Hierarchical comparisons and scoring are conducted sequentially from top to bottom within the objective tree, thereby establishing the pairwise comparison judgment matrix. Evaluation criteria are detailed in Table 2. The pairwise comparison judgment matrix for the first-level sub-objectives is shown in Table 3; the second-level sub-objective comparison matrix is derived similarly.

The initial and the corresponding normalized weigh coefficients (*W*_i_ and *W*_i_′) are calculated by Formulas (4) and (5), respectively. Unidirectional normalization is performed on the twenty-three sets of indicator data for each batch of particles to obtain the corresponding values *C*_i_. Multiply each *C*_i_ by its respective weigh, sum the results, and the comprehensive evaluation score GI is obtained according to Formula (6). A higher score indicates superior dissolvability and taste properties.

(4) $$W_{i}=\sqrt[{m}]{a_{i1}a_{i2}\cdots a_{\mathrm{im}}}$$

(5) $${W_{i}}^{\prime }=\frac{W_{i}}{\sum_{i=1}^{m}W_{i}}$$

(6) $$\mathrm{GI}=\sum_{i=1}^{m}{W_{i}}^{\prime }\times C_{i}$$

### 2.8. Statistical Analysis

The OriginPro 2025 (OriginLab Corporation, Northampton, MA, USA) was used to perform the first-derivative analysis and plotting of the turbidity curve and the particle size test *D*_50_ data. The principal component analysis (PCA) is a classical data reduction technique that transforms high-dimensional correlated variables into a small number of uncorrelated composite variables, known as principal components (PCs). PCA typically outputs two types of results: score plots reflect the distribution of samples in the principal component space, revealing similarities and differences among samples; loading plots are used to analyze the relationships between original variables and their contributions to the principal components [34]. PCA was performed using the SIMCA 14.1 (Sartorius Stedim Data Analytics AB, Umeå, Sweden). The latent class trajectory modeling (LCTM) was used to conduct longitudinal data analysis on the full-process results of multiple indicators for various granular products over time. LCTM represents an emerging methodology capable of simplifying heterogeneous research samples into homogeneous patterns or categories. These clusters or categories can potentially include random effects to account for individual variation within them. Based on an improved preliminary working model [35], the optimal number of categories was determined by testing *K* = 1~7. The selected number of categories was based on the minimum Bayesian Information Criterion (BIC). This method employs the maximum likelihood method, utilizing the ‘hlme’ function within the R software environment (Version 4.5.0) to fit the model for data analysis.

## 3. Results and Discussion

### 3.1. Static Descriptive Analysis

The static analysis means the characteristics at the end of the dissolving process (i.e., 5 min) were analyzed. These characteristics include *D*_10_, *D*_50_, *D*_90_ and *Span*, reflecting particle size distribution characteristics; the cumulative dissolution, measuring dissolution extent; approximate solubility, indicating soluble solids content; the turbidity, reflecting the clarity of a liquid; the taste values, representing flavor.

#### 3.1.1. Particle Size

**Figure 3:**
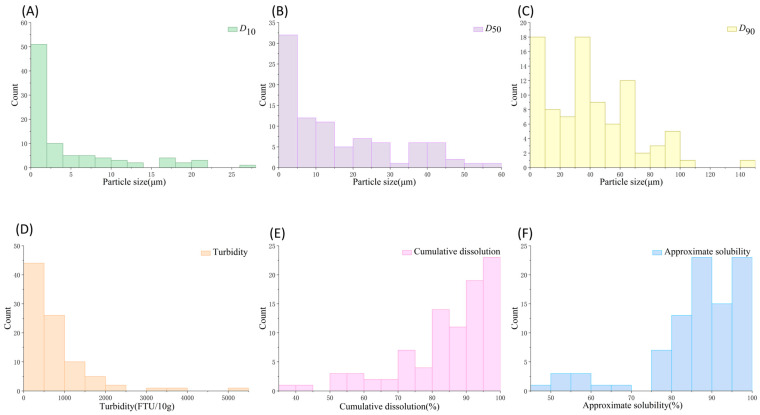
Frequency distribution of 6 dissolving end characteristics for 90 batches of plant/medicine extract granules. (**A**) *D*_10_; (**B**) *D*_50_; (**C**) *D*_90_; (**D**) Turbidity; (**E**) Cumulative dissolution; (**F**) Approximate solubility.

90 batches of granules underwent 300 s online measurements according to the method described in Section 2.2. Histograms of *D*_10_, *D*_50_, and *D*_90_ at the predefined dissolving endpoint are shown in Figure 3A, B, and C, respectively. *D*_10_ were ranged from 0.382 to 26.2 μm. The smallest *D*_10_ value was observed in the Yuye Jiedu Granule (No. 18), and the largest value was found in the Xiaoyan Tuire Granule (No. 7). The *D*_10_ ranges of Chinese herbal granules, Kampo granules, coffee granules, and self-made single-extract granules were 0.382–26.2 μm, 0.961–21.6 μm, 0.495–1.31 μm, and 0.469–11.5 μm, respectively. *D*_50_ values for 90 granules samples were ranged from 0.552 to 57.7 μm. The minimum *D*_50_ value was observed in the Yuye Jiedu Granule (No. 18), and the maximum in the Bawei Dihuang Pill Kampo Granule (No. 33). The *D*_50_ ranges of Chinese herbal granules, Kampo granules, coffee granules, and self-made single-extract granules were 0.552–45.7 μm, 4.05–57.7 μm, 0.943–12.8 μm, and 2.43–43.95 μm, respectively. *D*_90_ for 90 granules were ranged from 0.382 to 109 μm. The minimum value was observed in Yuye Jiedu Granule (No. 18), and the maximum in Xiaqinglong Tang Kampo Granule (No. 31). The *D*_90_ ranges of Chinese herbal granules, Kampo granules, coffee granules, and self-made single-extract granules were 0.943–136 μm, 27.1–109 μm, 3.59–63.0 μm, and 3.79–86.3 μm, respectively. 39 batches of granules, including 10 batches of Chinese herbal granules, 18 batches of Kampo granules, 10 batches of self-made single-extract granules, and 1 batch of coffee granule had *D*_90_ exceeding 40 μm, indicating the presence of visible insoluble particles. The *Span* values were ranged from 0.650 to 23.8. Only two batches granules with a *Span* value below 1.0, while 73 batches ranging from a *Span* of 1.0 to 5.0. All remaining samples with a *Span* value above 5.0. The minimum *Span* value was observed in Xiaoyan Tuire Granule (No. 7), and the maximum in Dashanzha Granule (No. 5). 15 batches of granules were with *Span* values above 5.0. The *Span* ranges of Chinese herbal granules, Kampo granules, coffee granules, and self-made single-extract granules were 0.653–23.8, 1.00–7.07, 1.70–5.37, 1.32–17.3, respectively.

The particle size distributions of the four types of granules exhibited certain differences, which were mainly attributed to factors such as raw material composition, formulation, preparation process, and excipients. Chinese herbal granules and Kampo granules are formulated with multiple Chinese medicinal herbs. Compared with self-made single-extract granules and coffee granules with simple formulations, they possess more complex herbal compositions and often contain polysaccharide-rich and mucilage-rich herbs such as *Astragalus membranaceus* [36], *Rehmannia glutinosa* [37], and *Ophiopogon japonicus* [38]. However, extracts rich in carbohydrates tend to have a low glass transition temperature, which can lead to increased stickiness and poor flowability after spray drying, thereby promoting particle aggregation and resulting in larger particle size [39]. Meanwhile, the excipients contained in the Chinese herbal granules in this study were mostly sucrose (excipient information shown in Table S1), which is also a traditional diluent. Due to its low glass transition temperature, the product powder may become sticky and exhibit poor flowability after the drying process [39]. In contrast, self-made single-extract granules are composed of only a single herb, with a relatively simple component system and weak synergistic effect of polysaccharides and viscous substances. Coffee granules are derived from roasted coffee bean extract, which does not contain high-viscosity polysaccharides, pectin, or mucilage commonly found in Chinese medicines, and their formulation is highly uniform and stable, leading to the overall smallest particle size and the narrowest distribution range. Based on the above analysis, the overall particle size distribution results indicated that coffee granules and self-made single-extract granules exhibited relatively small particle sizes, and coffee granules and Kampo granules have a narrower Span value range, meaning their particle size distribution is relatively uniform. Accordingly, the coffee granules could serve as the target for improving the particle size distribution of other plant-derived instant granules.

#### 3.1.2. Turbidity Results and Analysis

Following the method described in Section 2.3, online turbidity measurements were conducted to monitor the dissolving process of 90 batches of granular samples. The histogram of turbidity values at the 300 s time point for 90 batches of granules is shown in Figure 3D. According to the turbidity classification method previously defined by the research team [40], solution turbidity values in the range of 0–70 FTU indicate complete dissolving, 70–350 FTU indicate slight turbidity, 350–2000 FTU indicate turbidity, and >2000 FTU indicate severe turbidity. Results showed that among 90 batches of samples, the distribution of 300 s endpoint turbidity was as follows: 17 batches in 0–70 FTU, 19 batches in 70–350 FTU, 50 batches in 350–2000 FTU, and 4 batches exceeding 2000 FTU. Turbidity of Chinese herbal granules were ranged from 0 to 1713 FTU, where the highest turbidity was observed in Xiaoer Jingxing Zhike Granule (No. 27), and 10 samples such as Jianpi Shengxue Granule (No. 1) and Xuanmai Ganju Granule (No. 2), showed zero turbidity. The Kampo granules had the turbidity range of 365–5340 FTU, in which 18 batches were ranged from 350 to 2000 FTU, and 2 batches exceeded 2000 FTU. The Guizhi Fuling Pill Kampo Granule (No. 42) exhibited the highest turbidity, while the Fangji Huangqi Tang Kampo Granule (No. 43) showed the lowest. Coffee granules exhibited turbidity ranging from 394 to 1008 FTU. The Moccona coffee Granule (No. 56) showed the highest turbidity, while the Davidoff coffee Granule (No. 53) exhibited the lowest. The turbidity range of self-made single-extract granules was 0–3161 FTU, where the Polygonum Bistorta Granule (No. 78) exhibited the highest turbidity, and the Rehmanniae Radix Granule (No. 80) showed zero turbidity, attributed to its content of water-soluble components like carbohydrates, iridoids, phenylethanoid glycosides [41].

Overall, significant differences were observed in the turbidity at the 300 s endpoint among the four types of granules. Chinese herbal granules demonstrated the best performance, while Kampo granules and coffee granules showed poorer results. When conducting optimization studies on turbidity results for different types of granules, one may refer to Chinese herbal granules and improve the results by selecting appropriate excipients. These observed turbidity differences among the four granule types can be comprehensively explained by the nature of turbidity itself and the various factors affecting it. Turbidity is the result of the interaction between light and suspended particles in a liquid. Suspended particles scatter light, causing the liquid to appear turbid. Thus, turbidity characterizes the degree to which light transmission through a liquid is impeded, representing a physical parameter [42]. Distinct differences in turbidity exist among the four types of granules, which can be mainly attributed to raw material composition, manufacturing process, particle size, and excipients. For self-made single-extract granules, such as *Sophorae Flavescentis Radix* formula granules, alkaloids are the primary active constituents [43], most alkaloids are practically insoluble or sparingly soluble in water, which may account for their relatively high turbidity values. The extraction process of self-made single-extract granules is relatively uniform, typically consisting of water extraction, concentration, and drying. Some constituents extracted under boiling conditions are poorly re-dissolvable in hot water, leading to elevated turbidity in certain self-made single-extract granule products [44]. During water decoction of various Chinese medicinal herbs, insoluble macromolecular impurities—including resins, colloids, and plant proteins—are present in the extract and contribute to turbidity [45]. However, some Chinese herbal granules employ refining processes such as alcohol precipitation for impurity removal, resulting in high elimination rates of poorly soluble macromolecules [44]. Kampo formulations emphasize the quality of crude herbal materials and retain substantial amounts of raw herb powder and crude extracts, with persistent residues of numerous poorly soluble macromolecules, thereby increasing turbidity. Additionally, excipients used in Kampo granules often include water-insoluble components such as magnesium stearate, which may further contribute to their high turbidity values [44]. Turbidity depends not only on the presence of particles in solution but also on their size, number, shape, and surface area. During the dissolution of granules, these factors do not act independently but collectively influence turbidity measurement. Notably, the number of particles in the range of 10–100 μm exerts a considerable effect on turbidity, with *D*_10_ and *D*_50_ being particularly influential [46]. Ideal coffee grinding should produce a bimodal particle size distribution, with moderate proportions of both the coarse fraction and finer particles [47], which may influence the turbidity of the coffee beverage. Meanwhile, coffee brew as a whole, and its high-molecular-weight fraction (HMWF), was able to form emulsions with good physical stability and excellent oxidative stability [48]. All the emulsions exhibited a brown, opaque, and homogeneous appearance [49], which also contributes to the turbidity of coffee beverages.

#### 3.1.3. Cumulative Dissolution

The cumulative dissolution at each designed time point was calculated according to the method described in Section 2.4. The histogram of 5 min cumulative dissolution for 90 batches of granules is plotted in Figure 3E. It was seen that cumulative dissolution were ranged from 37.1% to 99.8%. The minimum cumulative dissolution at 5 min was observed in Polygoni Multiflori Radix Preparata Granule (No. 64), while the maximum was in Linggui Zhugan Tang Kampo Granule (No. 46). The Polygoni Multiflori Radix Preparata Granule exhibited constrained cumulative dissolution due to the poor water solubility of components like anthraquinones and polyphenols [50]. The cumulative dissolution ranges of Chinese herbal granules, Kampo granules, coffee granules, and self-made single-extract granules were 41.9–98.3%, 61.5–99.8%, 70.1–99.7%, 37.1–98.0%, respectively. The number of granules with cumulative dissolution exceeding 85% accounted for approximately 76.7%, 60%, 60%, and 43.3% in the aforementioned four types of granules, respectively. Most of Chinese herbal granules demonstrated the good dissolution performance. This is because most Chinese herbal granules are water-extracted, and the manufacturing process may include steps to remove impurities (such as macromolecules like polysaccharides or insoluble substances). The self-made single-extract granules exhibited the greatest variability of cumulative dissolution among batches.

#### 3.1.4. Approximate Solubility

The histogram of approximate solubility results for 90 batches of granules is shown in Figure 3F. Approximate solubility were ranged from 49.8% to 99.9%, with 38 batches exhibiting approximate solubility between 90% and 100%, including 14 batches of self-made single-extract granules, 23 batches of Chinese herbal granules, and 1 batch of coffee granule. 36 batches had approximate solubility between 80% and 90%, and they were 11 batches of self-made single-extract granules, 6 batches of Chinese herbal granules, 10 batches of Kampo granules, and 9 batches of coffee granules. The remaining 16 batches of granules had approximate solubility below 80%. The granule product with the highest approximate solubility was the Zhike Pipa Granule (No. 15), while the lowest was the Suanzaoren Tang Kampo Granule (No. 36).

Overall, approximate solubility results showed Chinese herbal granules performed best (most batches exceeding 90%), followed by self-made single-extract granules, while coffee granules and Kampo granules gave relatively lower approximate solubility. The preparation process of Chinese herbal granules underwent multiple refinement steps, resulting in high approximate solubility [51]. Coffee granules contained insoluble fiber content [44]. The Kampo granules with approximate solubility below 75% were produced by Kracie company, Tokyo, Japan, since these products used corn starch as excipients, which exhibited limited water solubility. Even under autoclaving at 121 °C, a considerable portion of corn starch remains undissolved as insoluble residues [52]. Therefore, after centrifugation at room temperature, the insoluble corn starch will deposit, which may account for the relatively low approximate solubility of this type of Kampo granules.

#### 3.1.5. Taste Value Measurement Results

**Figure 4:**
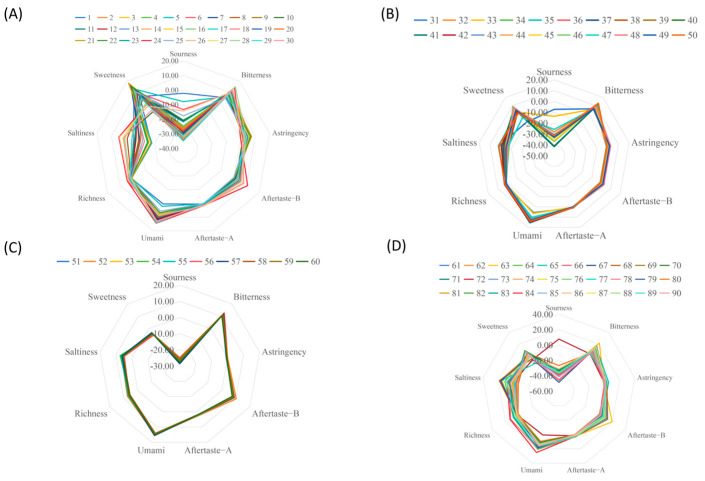

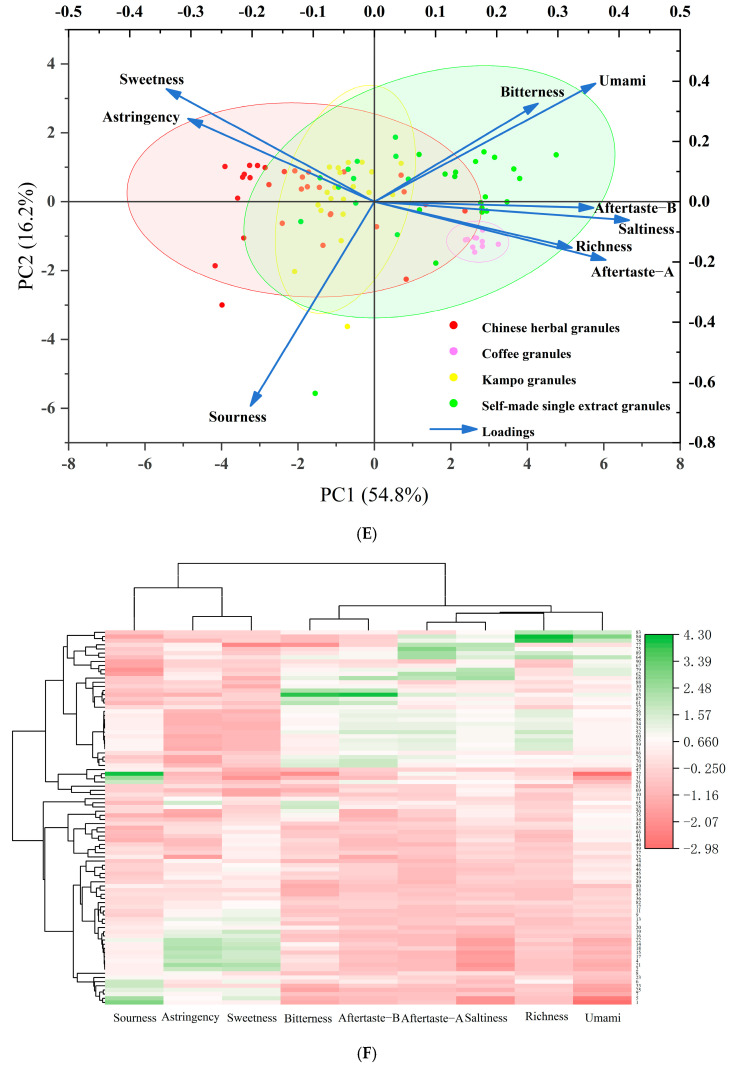
Multivariate analysis of e-tongue descriptors. (**A**) Taste for Chinese herbal granules; (**B**) Taste for Kampo granules; (**C**) Taste for coffee granules; (**D**) Taste for self-made single-extract granules; (**E**) The PCA biplot of 90 batches of granules’ taste data; (**F**) Heatmap of 90 batches of granules’ taste data.

The electronic tongue’s responses of sour, bitter, sweet, salty, and umami, were obtained using the method described in Section 2.6. The taste radar charts for Chinese herbal granules, Kampo granules, coffee granules, and self-made single-extract granules are shown in Figure 4A, B, C and D, respectively. The radar charts directly revealed the overall response profiles of the granules across different mouthfeel characteristics. Differences in the shape of the charts, provided intuitive evidence that the electronic tongue could effectively capture and distinguish the overall flavor fingerprints of different instant granules.

PCA was performed on matrix X (size 90 × 9) comprising 9 electronic tongue’s responses for 90 batches of granules. All variables were mean-centered and auto-scaled to ensure equal weighting., the results show that the first two principal components could explain 71% variation in the information from the original data. The PCA biplot is shown in Figure 4E, which reveals that the electronic tongue can clearly distinguish coffee granules from the other three types of granules. It could also separate most Chinese herbal granules and self-made single-extract granules, while the Kampo granules overlapped with other granules. The heatmap is furtherly introduced to analyze the relevant attributes of 90 batches of granules, as shown in Figure 4F. The heatmap indicated that Chinese herbal granules exhibited high sweetness intensity, while coffee granules and self-made single-extract granules possessed elevated umami and bitterness intensities. This finding aligned strongly with the PCA, collectively indicating that the overall taste profiles of Chinese herbal granules and Kampo granules were relatively more acceptable than those of self-made single-extract granules. This discrepancy may relate to excipients (e.g., masking agents) added to Chinese herbal granules and Kampo granules. In summary, the present findings demonstrated the capability of electronic tongue in distinguishing complex multi-component plant-derived granule samples, offering a time-effective technical pathway for objectively evaluating the taste quality of instant granules.

### 3.2. Dynamic Analysis of the Dissolving Process

#### 3.2.1. Dissolving Rate Characterization

The *T*_50_ of 90 batches of samples were ranged from 5 s to 220 s. The shortest *T*_50_ was observed in Nestlé Gold Coffee Granule (No. 60), while the longest was in Baihu Jia Renshen Tang Kampo Granule (No. 38). For Chinese herbal granules, Kampo granules, coffee granules, and self-made single-extract granules, the *T*_50_ ranges were 5–154 s, 6–220 s, 5 to 14 s and 5–135 s, respectively, and the mean of *T*_50_ were 38.7 s, 112.1 s, 7.21 s and 41.5 s, respectively. The *T*_e_ of 90 samples were ranged from 91 s to 501 s. The shortest *T*_e_ was observed in Suanzaoren Tang Kampo Granule (No. 36), while the longest was in Polygonum Bistorta Granule (No. 78). Except for Polygonum Bistorta Granule (No. 78) and Arecae Semen Carbonisatum Granule (No. 84, *T*_e_ = 410 s), all other samples reached turbidity equilibrium within 300 s. For Chinese herbal granules, Kampo granules, coffee granules, and self-made single-extract granules, the *T*_e_ ranges were 96–158 s, 91–266 s, 165–280 s and 114–501 s, respectively, and the mean of *T*_e_ were 126.6 s, 151.1 s, 230.1 s and 180.7 s, respectively. It could be found that the mean value of *T*_50_ decreased in the following order: Kampo granules > self-made single-extract granules > Chinese herbal granules > coffee granules. The mean value of *T*_e_ decreased in the following order: coffee granules > self-made single-extract granules > Kampo granules > Chinese herbal granules. Both *T*_e_ and *T*_50_, and *T*_e_ and final solution turbidity, were relatively independent indicators whose correlation coefficients were 0.0098 and 0.35, respectively. The coffee granules had relatively small particle sizes and uniform distribution at the end time of dissolving test, and their *T*_50_ values indicated fast dissolving speed. In contrast, the Kampo granules possessed relatively large particle sizes and wide distribution at the dissolving end, and their *T*_50_ values were long.

#### 3.2.2. Rehydration Behavior Classification

**Figure 5:**
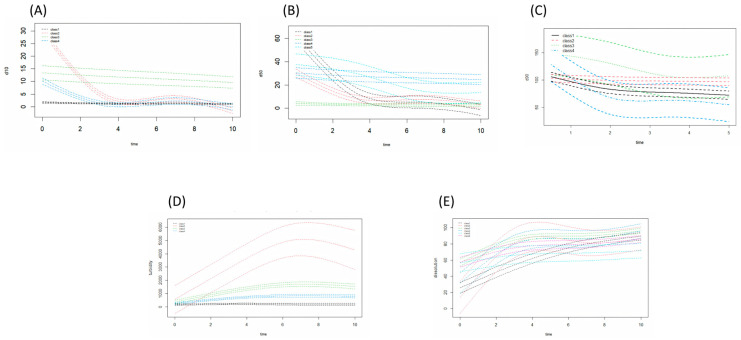
Latent class trajectory analysis of particle process indicators based on LCTM and their representative trajectory maps. (**A**) *D*_10_; (**B**) *D*_50_; (**C**) *D*_90_; (**D**) turbidity; (**E**) cumulative dissolution.

Following the method described in Section 2.8, the latent class trajectory modeling (LCTM) was performed to characterize heterogeneous patterns of *D*_10_, *D*_50_ and *D*_90_ values, turbidity, and cumulative dissolution data of 90 batches of granules over time points of 0.5 min, 1 min, 1.5 min, 2 min, 3 min, and 5 min. The resulting fitted plots are shown in Figure 5. Model fit statistics BIC values are presented in Table 4. The optimal number of categories fitted for *D*_10_ data is 4, with *D*_50_, *D*_90_, turbidity, and cumulative dissolution data being 5, 4, 4, and 6, respectively. The classification distribution of granules across different metrics is shown in the Supplementary Table S5. Among *D*_10_ trajectories, 48 granules belonged to Class 1, while 3, 37, and 2 particles belong to Classes 2–4, respectively. For *D*_50_ trajectories, 5 granules belonged to Class 1, while 9, 23, and 48 granules belong to Classes 2–5, respectively. For *D*_90_ trajectories, 4 granules belonged to Class 1, while 16, 13, and 57 granules belong to Classes 2–4, respectively. The turbidity trajectories included 45 granules classified as Class 1, with 3, 16, and 26 granules classified as Classes 2–4, respectively. The cumulative dissolution trajectories included 16 granules classified as Class 1, with 3, 14, 9, 17, and 31 granules classified as Classes 2–6, respectively.

The *D*_10_ trajectory classifications are as follows: (1) Class 1: Fine particle size decreases slowly during the initial 4 min before stabilizing, consistently remaining within the 0–5 μm range. (2) Class 2: Fine particle size rapidly decreases from approximately 30 μm to 0–5 μm within 0–4 min, then reaches equilibrium at around 4 min. (3) Class 3: Fine particle size exhibits a near-linear decrease between 0 and 5 min, with both initial and final particle sizes exceeding 10 μm. (4) Class 4: Fine particle size decreased from approximately 10 μm to 0–5 μm within 0–3 min, stabilizing after approximately 3 min. Overall performance showed Class 4 and Class 2 outperformed Class 1 and Class 3. Classes 2 and 4 accounted for a small proportion, and Class 1 generally outperformed Class 3.

The performance of each *D*_50_ trajectory classification is as follows: (1) Class 1: Median particle size decreased from approximately 60 μm to 5–10 μm within 0–4 min, reaching equilibrium around 4.5 min. (2) Class 2: Median particle size decreased from approximately 30 μm to 0–10 μm within 0–4 min, reaching equilibrium around 3.5 min. (3) Class 3: Median particle size decreased slowly during 0–4 min before stabilizing, remaining within the 0–5 μm range throughout. (4) Class 4: Median particle size exhibits an approximately linear continuous decrease from 0 to 5 min, with both initial and final particle sizes exceeding 20 μm. (5) Class 5: Median particle size gradually decreases from approximately 40 μm to about 15 μm between 0 and 5 min, with the rate of decrease diminishing over time. Overall performance indicated that Class 2 was superior to Class 3, which is superior to Class 1, which is superior to Class 5, which is superior to Class 4.

The performance of each *D*_90_ trajectory classification is as follows: (1) Class 1: Coarse particle size decreases from approximately 100 μm to about 75 μm within 0–2.5 min, then stabilizes after approximately 2.5 min. (2) Class 2: Coarse particle size exhibited a nearly linear continuous decrease from 0 to 5 min, with both initial and final particle sizes maintained at approximately 100 μm. (3) Class 3: Coarse particle size decreased from approximately 150 μm to approximately 100 μm between 0 and 3.5 min, reaching equilibrium after approximately 3.5 min. (4) Class 4: Coarse particle size decreased from approximately 125 μm to approximately 65 μm within 0–2.5 min, stabilizing after approximately 2.5 min. Overall performance indicated Class 4 outperformed Class 1, which outperformed Class 3, which outperformed Class 2.

The turbidity trajectories are classified as follows: (1) Class 1: Turbidity shows no significant change within 0–5 min, remaining consistently within the 0–500 FTU range. (2) Class 2: Turbidity rises continuously within 0–5 min, with endpoint values falling between 4000 and 5000 FTU. (3) Class 3: Turbidity steadily increases to 1000–2000 FTU within 0–5 min, with the rate of increase gradually slowing over time. (4) Class 4: Turbidity exhibits an approximately linear increase trend within 0–5 min, ultimately reaching approximately 800 FTU. Overall performance suggested that Class 1 was superior to Class 4, which was superior to Class 3, which was superior to Class 2.

The cumulative dissolution profiles for each classification are as follows: (1) Class 1: Dissolution exhibits near-linear increase within 0–5 min, reaching approximately 70%. (2) Class 2: Dissolution initially rises then declines within 0–5 min, ultimately stabilizing at approximately 90%. (3) Class 3: Dissolution increases from approximately 60% to approximately 90% within 0–5 min. (4) Class 4: Dissolution increases from approximately 30% to approximately 80% within 0–5 min. (5) Class 5: Dissolution increases from approximately 60% to approximately 70% within 0–5 min. (6) Class 6: Dissolution increases from approximately 60% to approximately 80% within 0–5 min. Overall, Class 2/3 outperformed Class 4/6, which in turn outperformed Class 1/5. However, the terminal dissolution values within each class differed slightly; classification primarily reflected the pattern of change during dissolution. Among the cumulative dissolution profiles of Class 2 trajectories, the UV-monitored apparent dissolution typically exhibited an initial rise followed by a decline. The corresponding samples included Chaihu Jia Longgu Muli Tang Kampo Granule (No. 35), and Guizhi Fuling Wan Granule (No. 42). It was estimated that complexes like supramolecular may form in Chaihu Jia Longgu Muli Tang Kampo Granule (No. 35) and Guizhi Fuling Wan Granule (No. 42), resulting in lowered UV absorption compared to free components [53].

In summary, the rehydration behavior demonstrated by multiple metrics across 90 batches of particles can be clearly described qualitatively. This indicates the diversity of granules dissolvability.

### 3.3. Principal Component Analysis

Combining the aforementioned dynamic and static analysis, a total of 23 indicators are organized as follows: cumulative dissolution at 0.5 min, 1 min, 1.5 min, 2 min, 3 min, and 5 min, respectively; approximate solubility, turbidity, *D*_10_, *D*_50_, *D*_90_, *Span*, *T*_50_, and *T*_e_; sourness, bitterness, astringency, sweetness, umami, saltiness, richness, aftertaste-B, and aftertaste-A response values. PCA was performed on matrix X (size 90 × 23) comprising 23 indicators for 90 batches of granules. All variables were mean-centered and auto-scaled to ensure equal weighting. The PCA results are shown in Table 5. Here, *R*^2^ indicates the contribution of the corresponding principal component number to the model, and *R*^2^_cum_ represents the cumulative contribution rate. The rule of thumb is to select the number of principal components that provide the best predictive capability for the PCA model. Simultaneously, eigenvalues greater than 1 were selected. Therefore, six principal components were chosen for the model. The cumulative *R*^2^ of the principal components is 81%, indicating that these six components can explain 81% of the information in the original data.

**Figure 6:**
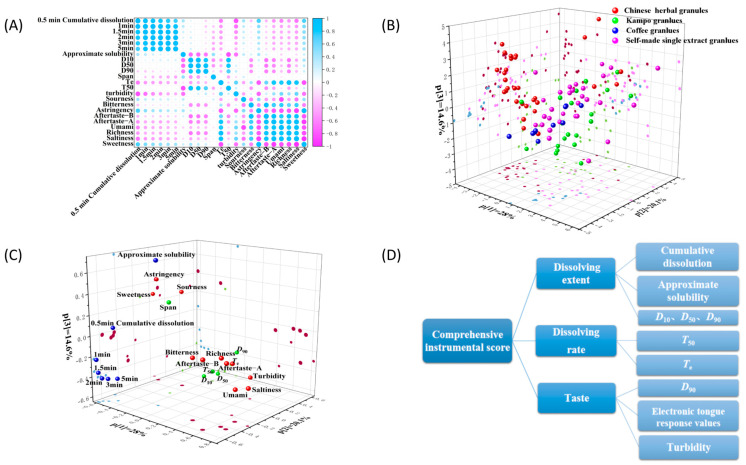
The multivariate analysis of dissolvability and taste properties of 90 batches of granules. (**A**) The correlation matrix of 23 quality attributes; (**B**) the 3D score plot of the PCA model; (**C**) the 3D loading plot of the PCA model(The solid three-dimensional dots in the plot represent original indicators, while the flat dots denote their projections onto the corresponding coordinate space); (**D**) the hierarchical dendrogram of the comprehensive instrumental score.

The correlation matrix for 23 variables is shown in Figure 6A. The red color indicates positive correlation, while the blue color indicates negative correlation. The closer the absolute value of the correlation coefficient is to 1, the larger the circle used to represent the correlation relationship becomes. The three-dimensional score plot and loading plot of the first three principal components of the PCA model are shown in Figure 6B and C, respectively. Figure 6B illustrates the spatial distribution patterns of 90 batches of granules, while Figure 6C visually identifies the primary variables governing this distribution. Results indicated that among the four kinds of granules, Chinese herbal granules and self-made single-extract granules exhibited greater separation along the PC1 axis. Meanwhile, Kampo granules and coffee granules overlapped to some extent in the spatial domain, suggesting they shared similar characteristics. The variables located on the same side of the PCA axis exhibited positive correlations, whereas those on the opposite side showed negative correlations. The results indicated that approximate solubility exhibited strong correlations with cumulative dissolution at various time points. Additionally, *D*_10_, *D*_50_ and *D*_90_ exhibited negative correlations with approximate solubility, featuring larger correlation coefficients. *T*_50_ was positioned on the opposite side of the *T*_e_, indicating a negative correlation between them. *D*_90_ correlated positively with turbidity, both reflecting the dissolving extent and indirectly influencing taste acceptance. Improving the dissolvability of instant granules was critical to the convenience and ability to dissolve effortlessly in water. The dissolving extent was closely associated with variables such as approximate solubility, cumulative dissolution at each time point, *D*_10_, *D*_50_, *D*_90_ and *Span*. The dissolving rate could be explained by *T*_50_ and *T*_e_. Taste was mainly characterized by e-tongue sensors, and turbidity and *D*_90_ could indicate a gritty feeling. Therefore, three sets of secondary indicators were established: dissolving extent, dissolving rate, and taste. Dissolving extent includes approximate solubility, cumulative dissolution at each time point, *D*_10_, *D*_50_, *D*_90_ and *Span* values. Dissolving rate encompassed *T*_50_ and *T*_e_. Taste was measured by turbidity, *D*_90_, and responses from electronic taste sensors. The hierarchical model for evaluating dissolvability and taste properties is shown in Figure 6D.

### 3.4. Comprehensive Evaluation of Dissolvability and Taste Properties

Based on the hierarchical model for evaluating dissolvability and taste properties of instant granules under Section 3.3, equal weights were given for the first three-level indexes, i.e., dissolving extent, dissolving rate and taste. Within each first-level index, the weights for the second-level indexes were ascertained by the pairwise comparison judgment matrix. Taking the second-level indexes belonging to the dissolving extent, for instance, the pairwise comparison judgment matrix is shown in Supplementary Table S6. Cumulative dissolution and approximate solubility were directly correlated with the dissolving extent of instant granules, and the two parameters were considered to have more importance. The particle sizes *D_1_*_0_, *D*_50_, and *D_9_*_0_ at the dissolving end shared equal weights and served as reference parameters, because the smaller the particles in the solution, the more completely the granules dissolved. The *Span* held relatively less importance in explaining the dissolving extent. Calculating the initial weight coefficient *W*_i_ using Formula (4) yielded *W*_1_ = (1 × 1 × 3 × 5)^1/4^ = 1.9680, *W*_2_ = 1.9680, *W*_3_ = 0.6560, and *W*_4_ = 0.3936, for approximate solubility, cumulative dissolution at each time point, *D*_10_, *D*_50_ and *D*_90_, *Span* values, respectively. Furthermore, the normalized weight coefficients *W*_i_′ were calculated using Formula (5). For example, *W*_1_′ = 1.9680/(1.9680 + 1.9680 + 0.6560 + 0.3936) = 0.3947. Similarly, *W*_2_′ = 0.3947, *W*_3_′ = 0.1316, *W*_4_′ = 0.0789. *T*_50_ and *T*_e_ were two second-level indexes belonging to the dissolving rate, and they were given equal importance. The normalized weight coefficients for *T*_50_ and *T*_e_ were both 0.5. Within the taste dimension, sweetness was a positive attribute in beverages. Conversely, bitterness was the primary negative factor affecting palatability. In addition, high turbidity and large *D*_90_ particle size may induce an unpleasant gritty or sandy texture. Acidity could mitigate the cloying sensation of sweetness, while umami enhanced flavor satisfaction against a sweet background, both being considered moderately important. Saltiness elevated flavor complexity, and astringency affected oral freshness, both ranking weak in importance. Aftertaste, however, played a relatively minor role in optimizing overall quality and flavor persistence. Based on the above analysis, the pairwise comparison judgment matrix for taste-related second-level indexes is shown in Supplementary Table S7. The normalized weight coefficients for turbidity, *D_9_*_0_, bitterness, sweetness, saltiness, umami, astringency, sourness, aftertaste-B, aftertaste-A, and richness were calculated as 0.1821, 0.1821, 0.1821, 0.1821, 0.0363, 0.0726, 0.0363, 0.0726, 0.0261, 0.0261, and 0.0261, respectively. Final weighting coefficients for 23 indicators were calculated by multiplying the weight values of the primary indicators by the normalized weight coefficients *W*_i_′ and were presented in Table 6. Although the alignment of the four distinct granule categories within a unified comprehensive score (GI), direct comparison of raw formulations between chemically unrelated products (such as coffee and therapeutic Kampo granules) lacks conventional sensory comparability, scaling their attributes under the unified AHP framework focuses exclusively on the shared physical principles of dissolution efficiency and sensor-based taste acceptability. The unified dataset prevents the model from overfitting to a single category and improves its generalization ability.

**Figure 7:**
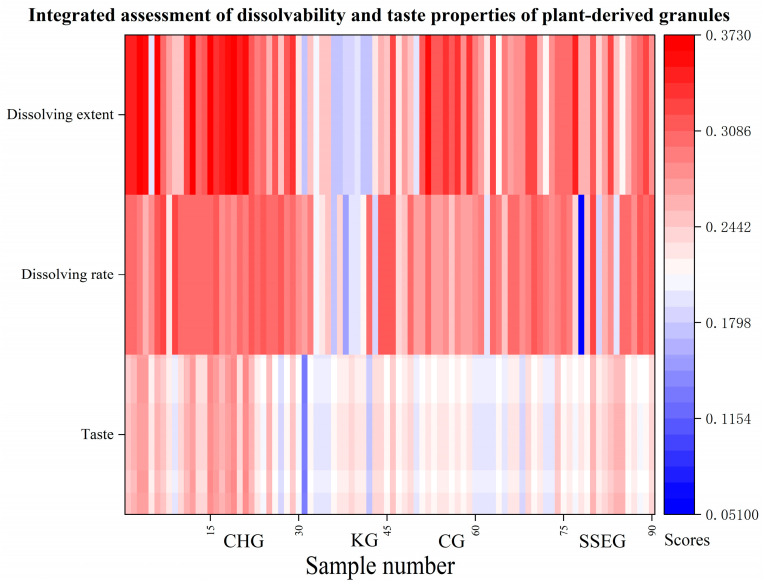
The evaluation panel of dissolvability and taste properties for 90 batches of plant-derived instant granules (CHG—Chinese herbal granules, KG—Kampo granules, CG—coffee granules, SSEG—self-made single-extract granules).

Before further analysis, all measured data were pretreated by the Min/Max method into the range [0, 1]. According to Formula (6), the preprocessed data of each indicator were multiplied by its normalized weight coefficient and summed to obtain the comprehensive score of dissolvability and taste properties for corresponding granule products. The results for all granules are provided in Supplementary Table S8. The evaluation panel of dissolvability and taste properties for 90 batches of instant granules is shown in Figure 7. 10 batches of instant granules scored above 0.9, 4 batches scored below 0.6, and the remaining 76 batches of granules clustered between 0.6 and 0.9. The granules with scores above 0.9 included: Sangju Ganmao Granule (No. 12), Qinghou Liyan Granule (No. 6), Xiasangju Granule (No. 19), Erding Granule (No. 3), Yuye Jiedu Granule (No. 18), Jingfang Granule (No. 21), Juhong Granule (No. 16), Jianpi Shengxue Granule (No. 1), and Xuanmai Ganju Granule (No. 2). The highest score of 0.9503 was achieved by Zhike Pipa Granule (No. 15). The granules with comprehensive evaluation scores below 0.6 points included: Xiaqinglong Tang Kampo Granule (No. 31), Suanzaoren Tang Kampo Granule (No. 36), and Baihu Jia Renshen Tang Kampo Granule (No. 38). The lowest score of 0.5406 was for Polygonum Bistorta Granule (No. 78).

The Polygonum Bistorta Granule (No. 78) had the lowest overall score of 0.5406, and its first-level indicator scores were 0.2635, 0.0514, and 0.2256 for the dissolving extent, dissolving rate, and taste, respectively. The dissolving rate and taste scores of the Polygonum Bistorta Granule were ranked in the middle among 90 batches, while the dissolving rate was scored as the lowest. This indicated that deficiencies existed across all three aspects, particularly with slow dissolving speed. The second-level indicators of approximate solubility, *Span*, *T*_e_, *T*_50_, and turbidity had notably low scores of 0.0690, 0.0202, 0.0000, 0.0514, and 0.0248, respectively. The approximate solubility of Polygonum Bistorta Granule was 76.06%, and the turbidity was 3161.2 FTU, at the end of dissolving. *T*_e_ and *T*_50_ were 501s and 200.5s, respectively. The *D*_50_–time curve and the turbidity–time curve during the dissolving process were classified as Class 3 and Class 2, respectively. This indicated that, except for the relatively favorable *D*_10_ and *D*_90_ profiles, other indicators showed below-average performance. Specifically, the decrease in fine-end particle size and median particle size was gradual, but both initial and final values remained low. Coarse-end particle size rapidly decreased to a low level. Turbidity continuously increased with a high endpoint value, and the cumulative dissolution endpoint value was relatively low. The poor taste and dissolvability of Polygonum Bistorta Granule primarily stemmed from high tannin and polysaccharide content [54,55]. This is compounded by high turbidity from plant residues. Through tannin removal, residue elimination, optimized extract formulation, spray-drying granulation, and minor formula adjustments, dissolvability can be enhanced, turbidity and equilibrium time reduced, particle size distribution improved, and overall quality elevated.

Zhike Pipa Granule (No. 15) had the highest overall score of 0.9503. The dissolving rate score of this granule was above average, but it received high marks for dissolving extent and taste. Among secondary indicators, Zhike Pipa Granule achieved the highest scores for turbidity and approximate solubility, with a favorable particle size score, though its umami and astringency scores were relatively low. Its rehydration behavior trajectory was classified as class 1, class 1, class 3, class 1, and class 6, respectively. This indicated moderate performance in *D*_50_, *D*_90_ and cumulative dissolution profiles, specifically: gradual decreases in fine and median particle sizes with low initial and final values; prolonged *T*_e_ and high endpoint for coarse particle size; minimal turbidity fluctuations with a low endpoint; and a moderate cumulative dissolution endpoint.

In addition to the granules with higher or lower scores mentioned above, class 4, which performed well on the *D*_10_ trajectory but accounted for a small proportion, also included Cirsii Japonici Herba Granule (No. 68). For class 2 with favorable *D*_50_ trajectories, class 1 with a smaller proportion, class 1 with a smaller proportion of *D*_90_ trajectories, class 2 with a smaller proportion of turbidity, and class 2 with a smaller proportion of dissolving rate, this also includes multiple batches of granules, such as Dashanzha Granule (No. 5), Notopterygii Rhizoma et Radix Granule (No. 61), etc. The same analysis applied to these granules revealed that their rehydration behavior trajectory classifications align well with their overall evaluation scores and the performance of secondary indicators within the system. This indicated that the qualitative results of rehydration behavior trajectory classification substantially validate the rationality of the constructed objective, quantitative evaluation system.

For Chinese herbal granules, Kampo granules, coffee granules, and self-made single-extract granules, the scores ranges were 0.7174–0.9503, 0.5687–0.8857, 0.7714–0.8805, and 0.5406–0.8961, and the means of scores were 0.8603, 0.6806, 0.8150, and 0.7791, respectively. The descriptive mean of the comprehensive scores revealed distinct adaptability profiles within the established framework, decreasing in the order of Chinese herbal granules > coffee granules > self-made single-extract granules > Kampo granules. This rank primarily reflects how well the multi-attribute indicators (dissolution and taste) are balanced within each specific product matrix under the standardized framework, rather than implying a cross-category quality superiority in real-world consumption. The score ranges for the dissolving extent of Chinese herbal granules, Kampo granules, coffee granules and self-made single-extract granules are as follows: 0.2000–0.3725, 0.1691–0.3204, 0.2840–0.3553, and 0.2127–0.3433, and the means of scores were 0.3155, 0.2185, 0.3181, and 0.2810, respectively. The score ranges for the dissolving rate of Chinese herbal granules, Kampo granules, coffee granules and self-made single-extract granules are as follows: 0.2282–0.3282, 0.1529–0.3177, 0.2504–0.3024, and 0.0514–0.3220, and the means of scores were 0.2988, 0.2457, 0.2747, and 0.2746. The score ranges for the taste of Chinese herbal granules, Kampo granules, coffee granules and self-made single-extract granules are as follows: 0.1865–0.2855, 0.1321–0.247, 0.2001–0.2475, 0.2001–0.2309, and 0.1887–0.2625, and the means of scores were 0.2461, 0.2164, 0.2223, and 0.2234, respectively. Based on the results, Chinese herbal granules performed best in terms of overall quality, dissolving rate, and taste, and demonstrated the most consistent overall performance; coffee granules stood out in terms of dissolving extent, with overall quality second only to Chinese herbal granules; self-made single-extract granules performed at an average level overall, but showed poor consistency in dissolving rate; Kampo granules ranked lowest across all evaluation criteria, with significant room for improvement in both dissolvability and taste. The four types of granules presented distinct overall dissolvability and taste characteristics. Based on their strengths and weaknesses, specific areas for improvement can be further identified. Furthermore, by cross-referencing the technical strengths between categories—such as transferring the advanced taste-masking strategies of Chinese herbal granules and the rapid rehydration microstructure design of coffee granules—a generalized roadmap for processing optimization and formulation refinement across diverse instant granule systems can be effectively established.

### 3.5. Model Evaluation and Application

**Figure 8:**
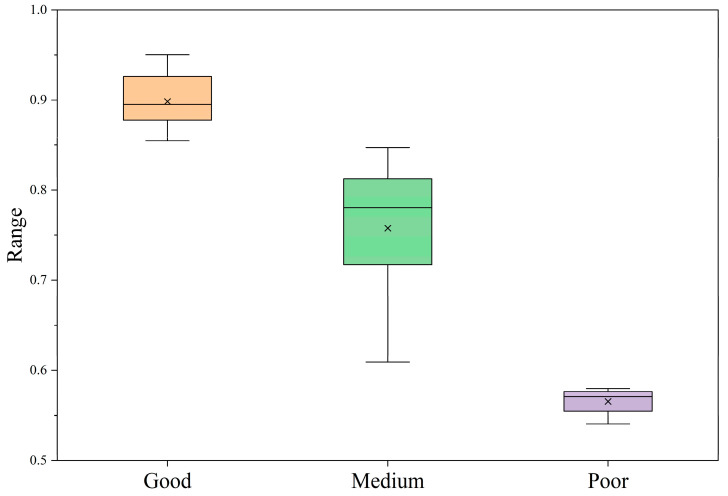
The box plot of comprehensive scores for each grade.

The evaluation method established in this study is intended to classify samples into corresponding quality grades. Specifically, samples were divided into three grades (Good, Medium, Poor) using 0.6 and 0.85 as classification thresholds. As shown in Figure 8, the box plot of comprehensive scores for each grade indicated obvious separation among the three groups: the median scores decreased sequentially from Good to Medium to Poor, with low overlap between groups. The results of one-way analysis of variance (ANOVA) showed significant differences among the three grades (*p* < 0.05), demonstrating that the established evaluation system has excellent discriminative ability and can effectively identify samples with different quality levels.

**Figure 9:**
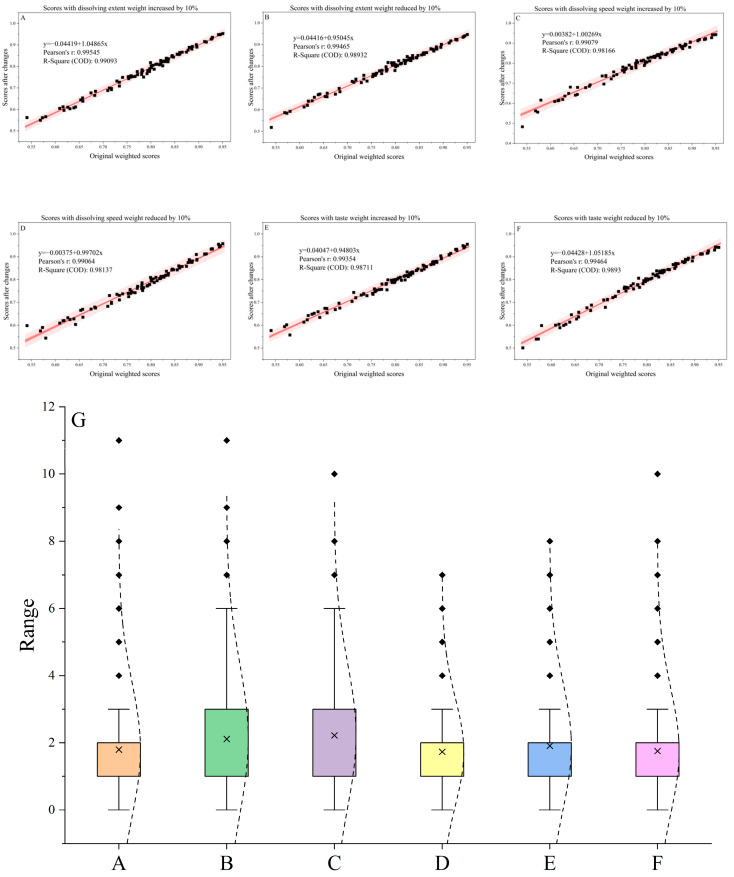

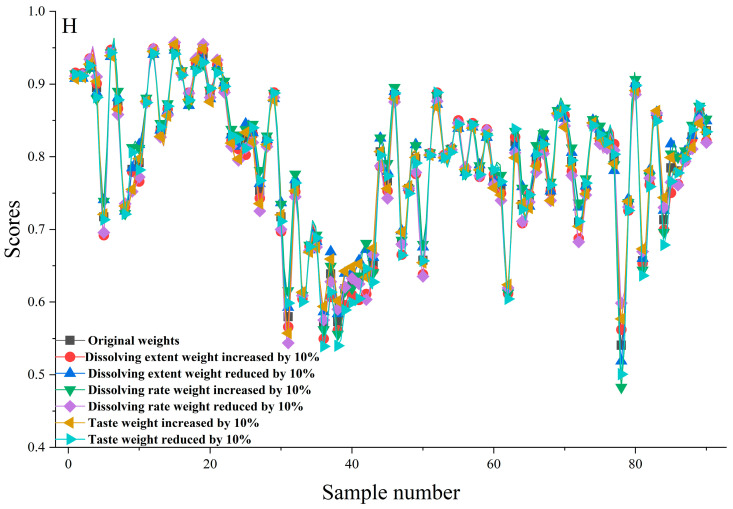
Model Sensitivity Analysis. (**A**) The scores obtained after +10% adjustment of the weights of dissolving extent; (**B**) the scores obtained after −10% adjustment of the weights of dissolving extent; (**C**) the scores obtained after +10% adjustment of the weights of dissolving rate; (**D**) the scores obtained after −10% adjustment of the weights of dissolving rate; (**E**) the scores obtained after +10% adjustment of the weights of taste; (**F**) the scores obtained after −10% adjustment of the weights of taste; (**G**) the boxplot of ranking fluctuations of samples under the six weight perturbation conditions(panels (**A**)–(**F**) correspond to the aforementioned Figure (**A**)–(**F**)); (**H**) the parallel trend plot of scores of the 90 batches under the seven conditions.

Meanwhile, to verify the robustness of the evaluation model, a sensitivity analysis was conducted by individually perturbing the weights of the three primary indicators by ±10%. The results showed that the average Spearman rank correlation coefficient between the scores of the 90 batches of granules under weight perturbation and the original scores was 0.9933, indicating high consistency in the ranking results. As shown in Figure 9A–F, after adjusting the weights of the three indicators (dissolution extent, dissolution rate, and taste) by ±10%, respectively, linear fitting was performed between the obtained scores and the original comprehensive scores. All groups showed good linear correlation, confirming that the scoring results had reasonable stability. When samples were classified into three grades (Superior, Moderate, Inferior) using 0.6 and 0.85 as thresholds, the grade retention rate of the 90 batches of granules under different weight perturbation conditions was 91.1%. Figure 9G shows the box plot of sample ranking fluctuations under six weight perturbation conditions, where columns A–F correspond to the six weight perturbation conditions shown in Figure 9A–F, respectively. Only three batches of samples had a ranking change exceeding 10 positions, namely Jianwei Xiaoyan Granule (No. 9), Jacobs Coffee Granule (No. 59), and Rosae Laevigatae Fructus Granule (No. 85). The average score differences between these three batches and their adjacent-ranked samples in the original weight system were 0.00165, 0.00185, and 0.00465, respectively, all below 0.01, indicating that these granules had relatively similar quality levels to their adjacent samples. In addition, the root-mean-square errors (RMSE) between the scores under the six perturbation schemes and the original scores were almost all less than 0.05, with median values of 0.00626, 0.00664, 0.00941, 0.00959, 0.00608, and 0.00587, respectively. Among the 90 batches of samples, only 1 batch had a score standard deviation greater than 0.03 across the seven scenarios (original weight plus six perturbations), while the remaining 89 batches all had a score standard deviation below 0.03. Figure 9H presents the parallel trend plot of the scores of the 90 batches under the seven conditions, from which it can be seen that the overall scoring trend remained basically consistent when the weights fluctuated within a reasonable range (±10%).

In summary, the comprehensive evaluation model established in this study has good robustness, is insensitive to small fluctuations in indicator weights, and yields relatively stable and reliable evaluation results, providing certain basic support for the quality evaluation and classification of granules.

## 4. Conclusions

This study developed an integrated multi-attribute evaluation framework focusing on dissolvability and taste properties for instant granules, achieving key advancements: (1) A novel methodology combining rehydration dynamics analysis with electronic tongue profiling has been established, providing a comprehensive assessment of both dynamic and static dissolvability and taste characteristics. (2) The weighted evaluation system developed through AHP provides a practical tool for quality control and formulation improvement of instant granules. The framework demonstrated potential for standardization of instant granule evaluation across product categories, addressing a critical gap in current quality assessment practices. Future research should focus on expanding the sample diversity, incorporating additional dimensions, and validating the framework in industrial settings.

Although this study has preliminarily established a comprehensive evaluation method for granules focusing on dissolvability and taste properties, excipients as potential interfering factors still need further research on their influences on granule dissolvability and taste characteristics, which offers a new direction for future investigations. Currently, relevant studies concerning the effects of excipients on granule dissolvability and taste properties are still insufficient, restricting in-depth discussion on this topic in the present work. Follow-up research will center on excipients and systematically explore their action mechanisms affecting granule dissolvability and taste performance, combined with the physicochemical features of different excipients. Further exploration will effectively enhance the completeness and scientific rationality of this evaluation system and lay a solid theoretical foundation for quality control and technological optimization of instant granules. This study did not include validation with human sensory panels, which can be further improved in future research. This framework was applied to plant extract granules. However, given the general applicability of its methodology, it can be reasonably extended to other similar food powder systems, such as dairy powder and protein powder.

## Figures and Tables

**Table 1:** Array performance of the electronic tongue sensor.

Sensor Name	Initial Taste	Aftertaste
CA0	Sourness	-
C00	Bitterness	Aftertaste-B
AE1	Astringency	Aftertaste-A
AAE	Umami	-
CT0	Saltiness	-
GL1	Sweetness	-

**Table 2:** Target tree diagram scoring criteria for each level.

Comparative Scoring	Relative Importance	Explanation
1	Equally important	Both contribute equally to the goal
3	Slightly important	Based on experience, one evaluation is slightly more favorable than the other
5	Fundamentally important	One evaluation is more favorable than the other based on experience
7	Certainly important	Based on experience, one evaluation proves more favorable than the other and has been demonstrated in practice
9	Absolutely important	Significantly greater in importance
(2, 4, 6, 8)	Median value of adjacency degree	Use it when a compromise is needed

**Table 3:** First-level sub-objective pairwise comparison judgment priority matrix.

	Dissolving Extent	Dissolving Rate	Taste
Dissolving extent	*a*_11_	*a*_12_	*a*_13_
Dissolving rate	*a*_21_	*a*_22_	*a*_23_
Taste	*a*_31_	*a*_32_	*a*_33_

**Table 4:** Fit statistics for trajectory class models.

*K*	BIC				
	*D*_10_	*D*_50_	*D*_90_	Turbidity	Cumulative Dissolution
1	2832	3921.31	4436	7960	4090
2	2240	3650	4128	7644	3919
3	2087	3573	4073	7510	3851
4	2019	3544	4068	7481	3816
5	2025	3523	4082	7486	3820
6	2046	3532	4069	7513	3809
7	2050	3549	4104	7540	3834

**Table 5:** Eigenvalues and explained variance of principal components.

Number of PC	Eigenvalue	*R*^2^ (%)	*R*^2^_cum_ (%)
1	35.8	28.0	28.0
2	21.7	20.1	48.1
3	3.36	14.6	62.7
4	1.62	7.03	69.7
5	1.45	6.31	76.0
6	1.16	5.04	81.0

**Table 6:** The comprehensive evaluation framework for dissolvability and taste properties of plant extract instant granules.

Primary Indicator	Weight of Primary Indicator (%)	Secondary Indicator	Weight of Secondary Indicator (%)
Dissolving extent	33.33	Approximate solubility	13.16
		Cumulative dissolution	13.16
		Particle sizes (*D*_10_, *D*_50_, *D*_90_)	4.39
		*Span*	2.63
Dissolving rate	33.33	The half-life of granules dissolving (*T*_50_)	16.67
		The time to turbidity equilibrium (*T*_e_)	16.67
Taste	33.33	Turbidity	6.07
		Particle size *D*_90_	6.07
		Bitterness	6.07
		Sweetness	6.07
		Saltiness	1.21
		Umami	2.02
		Astringency	1.21
		Sourness	2.02
		Aftertaste-B	0.87
		Aftertaste-A	0.87
		Richness	0.87

## Data Availability

The original contributions presented in the study are included in the article/supplementary material, further inquiries can be directed to the corresponding authors.

## Supplementary Materials

The following supporting information can be downloaded at: https://www.mdpi.com/article/10.3390/foods15112000/s1, Table S1: Information of 30 batches of Chinese herbal granules; Table S2: Information for 20 batches of Kampo granules; Table S3: Information of 10 batches of coffee granules; Table S4: Information of 30 Batches of self-made single extract granules; Table S5: Classification results of process trajectory classification based on five time dependent indicators for different granules; Table S6: The dissolving extent indicator second-level sub-objective pairwise comparison judgment optimality matrix; Table S7: The taste second-level sub-objective pairwise comparison judgment optimality matrix; Table S8: The evaluation scores for different granules.
