# Supplementary material for: A Comprehensive Instrumental Analysis Framework for Assessing the Dissolvability and Taste Properties of Plant Extract Instant Granules
Source: Foods. 2026 Jun 3;15(11):2000. doi: 10.3390/foods15112000 (PMC13257170; doi:10.3390/foods15112000)
Supplement: Supplementary file 1 [file foods-15-02000-s001.zip › foods-4238194-supplementary.pdf]

Supplementary Materials

Table S1 Information of 30 batches of Chinese herbal granules

No.	Name	Oral dose/g	Excipients	Batch No.
1	Jianpi Shengxue granules	15	sucrose, vitamin C, citric acid	Z10940043
2	Xuanmai Ganju granules	40	sucrose, dextrin	180505
3	Er Ding granules	60	sucrose	1803023
4	Qingre Qushi granules	30	sucrose	180607
5	Dashanzha granules	45	sucrose , citric acid	180812
6	Qinghou Liyan granules	30	sucrose	0780876
7	Xiaoyan Tuire granules	40	sucrose, corn starch	180506
8	Xiaoer Ganmao granules	48	sucrose, dextrin	17113033
9	Jianwei Xiaoyan granules	60	sucrose, dextrin	18122519
10	Xingbei Zhike granules	12	dextrin, aspartame	240119
11	Pugongying granules	45	sucrose, dextrin	01240123
12	Sangju Ganmao granules	33	sucrose, dextrin	202212028
13	Fufang Banlangen granules	45	sucrose, starch	L23K177
14	Tongxuan Lifei granules	18	sucrose	12240109
15	Zhike Pipa granules	30	sucrose	2303011
16	Juhuang granules	22	sucrose, dextrin	04231204
17	Wushicha granules	12	sucrose	240226
18	Yuyè Jiedu granules	36	sucrose	231111
19	Xiasangju granules	30	sucrose	VW30001
20	Xiaoqinglong granules	39	sucrose	231127
21	Jingfang granules	45	sucrose	240114
22	Yinqiao Jiedu granules	45	sucrose, dextrin	240202
23	Lanhua Qingwen granules	18	sucrose	A2301280H
24	Jinhua Qinggan granules	15	sucrose	24010501
25	Guanhuangmu granules	27	sucrose	04220104
26	Shaoma Zhijing granules	22.5	sucrose	230807
27	Xiaoer Jingxing Zhike granules	5	β-cyclodextrin, sucrose, stevioside, povidone K30, orange flavor	231208

No.	Name	Oral dose/g	Excipients	Batch No.
28	Sanhan Huashi granules	60	dextrin, aspartame	221234
29	Kunxinning granules	18	sucrose	230502
30	Huashi Baidu granules	20	sucrose	J2212070

Table S2 Information for 20 batches of Kampo granules

No.	Name	Manufacturer	One-day dose/g	Daily extract amount/g	Excipients	Batch No.
31	Xiaoqinglong Tang granules	KRAICRE	6.0	5.2	hydroxypropyl cellulose, lactose, polyoxyethylene, polyoxypropylene glycol	14Q527
32	Huanglian Jiedu Tang granules	KRAICRE	6.0	1.4	hydroxypropyl cellulose, lactose	13Q527
33	Bawei Dihuang Pill granules	KRAICRE	6.0	5.2	hydroxypropyl cellulose, lactose	26Q52X
34	Chaihu Guizhi Tang granules	KRAICRE	3.0		hydroxypropyl cellulose, lactose	25Q52X
35	Chaihu Jia Longgu Muli Tang granules	KRAICRE	6.0	3.9	hydroxypropyl cellulose, lactose	51Q527
36	Suanzaoren Tang granules	Sanwa Yakuhin	6.0		lactose, Corn Starch	DLD07
37	Guizhi Jia Gegen Tang granules		6.0		lactose, Corn Starch	DLD02
38	Baihu Jia Renshen Tang granules	Sanwa Yakuhin	6.0		lactose, Corn Starch	DGD01
39	Jingjie Lianqiao Tang granules	Sanwa Yakuhin	6.0		lactose, Corn Starch	DCD01
40	Chaihu Jia Longgu Muli Tang granules	Sanwa Yakuhin	6.0		lactose, Corn Starch	DEV02A
41	Guizhi Jia Longgu Muli Tang granules	Sanwa Yakuhin	6.0		lactose, Corn Starch	DID02A
42	Guizhi Fuling Pill granules	Sanwa Yakuhin	4.5	2.6	lactose, cellulose, calcium stearate, anhydrous silicic acid, partial α-amylase	DCD01
43	Fangji Huangqi Tang granules	Sanwa Yakuhin	6.0		lactose, Corn Starch	DID02
44	Huanglian Jiedu Tang granules	Tsumura	7.5	1.5	magnesium stearate, lactose hydrate	U26721
45	Guizhi Jia Zhufu Tang granules	Tsumura	7.5	3.75	magnesium stearate, lactose hydrate	U05301
46	Linggui Zhugan Tang granules	Tsumura	7.5	1.5	anhydrous light silica, magnesium stearate, lactose hydrate	U26751
47	Gouteng San granules	Tsumura	7.5	1.5	magnesium stearate, lactose	U46061

No.	Name	Manufacturer	One-day dose/g	Daily extract amount/g	Excipients	Batch No.
					hydrate	
48	Guizhi Tang granules	Tsumura	7.5	3.0	magnesium stearate, lactose hydrate	W00071
49	Wenqing Yin granules	Tsumura	7.5	3.75	magnesium stearate, lactose hydrate	U46401
50	Chaihu Guizhi Tang granules	Tsumura	7.5	4.0	magnesium stearate, lactose hydrate, sucrose fatty acid ester	U33481

Table S3 Information of 10 batches of coffee granules

No.	Name	Batch No.	No.	Name	Batch No.
51	Mosswell House Coffee granules	20230401	56	Moccona Coffee granules	20230103
52	Casino Agglomere Classique Coffeegranules	20220920	57	AGF Coffee granules	20230701
53	DAVIDOFF Coffee granules	20221006	58	Vittoria Coffee granules	20230920
54	TASOGARE coffee granules	20220813	59	JACOBS MONARCH Coffee granules	2300601
55	Ucc THE BLEND NO.114 Coffee granules	20230718	60	Nestlé Gold Coffee granules	231018

Table S4 Information of 30 Batches of self-made single extract granules

No.	Name	Batch No.	No.	Name	Batch No.
61	Notopterygii Rhizoma et Radix granules	P200518-623400-17A	76	Corydalis Bungeanae Herba granules	SGC201709-3
62	Cinnamomi Cortex granules	YP180209-526100-07A	77	Corydalis Bungeanae Herba granules	P190514-071200-02A
63	Scutellariae Radix granules	YP170912-276400-01A	78	Polygonum bistorta granules	P190320-273400-18A
64	Polygoni Multiflori Radix Preparata granules	PYP170919-617100-21A	79	Pogostemonis Herba granules	180209-529600-13A
65	Chuanxiong Rhizoma granules	YP170122-748411-01A	80	Rehmanniae Radix granules	180502-454850-02A
66	Atractylodis Rhizoma Stir-fried with Bran granules	PYP180208-024200-06A	81	Asari Radix et Rhizoma granules	1804297-113200-38A
67	Ligustici Rhizoma et Radix granules	P190116-113301-02A	82	Siegesbeckiae Herba granules	P190221-444200-02A
68	Cirsii Japonici Herba granules	P190419-068350-03A	83	Buddlejae Flos granules	P190424-638300-02A

No.	Name	Batch No.	No.	Name	Batch No.
69	Sinomenii Caulis granules	P190419-441000-01A	84	Arecae Semen Carbonisatum granules	PJ190617-537000-01A
70	Glycyrrhizae Radix et Rhizoma Preparata cum- Melle granules	180421-015200-01A	85	Rosae Laevigatae Fructus granules	P200608-333100-02A
71	Angelicae Sinensis Radix granules	YP170122-748304-02A	86	Visci Herba granules	P190226-134100-01A
72	Mume Fructus granules	J180606-672600-07A	87	Curculiginis Rhizoma granules	P190521-621100-03A
73	Sophorae Tonkinensis Radix et Rhizomat granules	P190521-532800-03A	88	Sophorae Flavescentis Radix granules	180209-046300-07A
74	Asparagi Radix granules	P190116-674200-03A	89	Menthae Haplocalycis Herba granules	YP180209-277700-13 A
75	Polygoni Avicularis Herba granules	P190522-463200-03A	90	Phragmitis Rhizoma granules	P190603-071200-03A

Table S5 Classification results of process trajectory classification based on five time dependent indicators for different granules

No.	D_{10} curve	D_{50} curve	D_{90} curve	Turbidity curve	Dissolution curve
1	Class1	Class4	Class2	Class1	Class6
2	Class1	Class2	Class2	Class1	Class6
3	Class1	Class2	Class3	Class1	Class3
4	Class2	Class1	Class3	Class1	Class6
5	Class1	Class1	Class1	Class1	Class5
6	Class1	Class3	Class3	Class1	Class6
7	Class3	Class4	Class4	Class1	Class5
8	Class3	Class4	Class4	Class1	Class6
9	Class3	Class4	Class4	Class1	Class6
10	Class1	Class3	Class4	Class1	Class1
11	Class3	Class4	Class4	Class1	Class6
12	Class1	Class2	Class3	Class1	Class6
13	Class3	Class4	Class4	Class1	Class5
14	Class3	Class4	Class4	Class1	Class3
15	Class1	Class1	Class3	Class1	Class6
16	Class3	Class4	Class2	Class1	Class6
17	Class3	Class1	Class1	Class1	Class5

No.	D_{10} curve	D_{50} curve	D_{90} curve	Turbidity curve	Dissolution curve
18	Class2	Class1	Class3	Class1	Class6
19	Class2	Class2	Class3	Class1	Class3
20	Class1	Class4	Class2	Class1	Class3
21	Class1	Class2	Class3	Class1	Class6
22	Class1	Class3	Class3	Class1	Class5
23	Class3	Class4	Class4	Class1	Class3
24	Class1	Class4	Class4	Class4	Class3
25	Class1	Class4	Class4	Class1	Class5
26	Class3	Class4	Class4	Class4	Class3
27	Class1	Class4	Class4	Class3	Class6
28	Class1	Class3	Class2	Class1	Class1
29	Class4	Class4	Class4	Class1	Class5
30	Class1	Class3	Class2	Class4	Class5
31	Class3	Class5	Class1	Class2	Class1
32	Class3	Class4	Class4	Class1	Class4
33	Class3	Class4	Class4	Class4	Class1
34	Class3	Class4	Class4	Class3	Class2
35	Class3	Class4	Class4	Class3	Class2
36	Class3	Class4	Class4	Class4	Class3
37	Class3	Class4	Class4	Class4	Class5
38	Class3	Class4	Class4	Class4	Class3
39	Class3	Class4	Class4	Class1	Class6
40	Class3	Class4	Class4	Class4	Class3
41	Class3	Class4	Class4	Class3	Class6
42	Class3	Class4	Class4	Class2	Class2
43	Class3	Class4	Class4	Class1	Class5
44	Class1	Class4	Class4	Class4	Class1
45	Class1	Class4	Class4	Class3	Class1
46	Class1	Class3	Class4	Class4	Class4

No.	D_{10} curve	D_{50} curve	D_{90} curve	Turbidity curve	Dissolution curve
47	Class1	Class5	Class1	Class3	Class1
48	Class3	Class5	Class4	Class4	Class1
49	Class1	Class4	Class4	Class4	Class1
50	Class3	Class4	Class4	Class3	Class1
51	Class1	Class3	Class4	Class3	Class4
52	Class1	Class3	Class4	Class4	Class5
53	Class1	Class3	Class4	Class1	Class6
54	Class1	Class3	Class4	Class4	Class5
55	Class1	Class3	Class4	Class4	Class4
56	Class1	Class3	Class4	Class3	Class4
57	Class1	Class3	Class4	Class4	Class4
58	Class1	Class3	Class2	Class3	Class4
59	Class1	Class3	Class4	Class4	Class4
60	Class1	Class4	Class4	Class4	Class3
61	Class1	Class2	Class2	Class4	Class6
63	Class3	Class5	Class4	Class3	Class1
63	Class1	Class2	Class2	Class1	Class3
64	Class3	Class4	Class4	Class1	Class5
65	Class3	Class4	Class4	Class1	Class3
66	Class3	Class4	Class4	Class1	Class4
67	Class3	Class4	Class2	Class4	Class6
68	Class4	Class2	Class3	Class4	Class3
69	Class1	Class3	Class3	Class1	Class6
70	Class1	Class3	Class4	Class1	Class6
71	Class3	Class4	Class4	Class1	Class6
72	Class3	Class4	Class4	Class1	Class6
73	Class3	Class4	Class4	Class1	Class1
74	Class3	Class4	Class4	Class1	Class6
75	Class1	Class3	Class4	Class3	Class6

No.	D_{10} curve	D_{50} curve	D_{90} curve	Turbidity curve	Dissolution curve
76	Class1	Class3	Class2	Class3	Class1
77	Class1	Class4	Class2	Class4	Class6
78	Class1	Class3	Class4	Class2	Class1
79	Class3	Class5	Class2	Class4	Class5
80	Class1	Class3	Class4	Class1	Class6
81	Class3	Class4	Class4	Class4	Class6
82	Class3	Class4	Class4	Class3	Class6
83	Class1	Class2	Class3	Class1	Class6
84	Class1	Class3	Class2	Class3	Class5
85	Class1	Class4	Class4	Class4	Class5
86	Class1	Class4	Class2	Class4	Class5
87	Class1	Class3	Class3	Class1	Class1
88	Class1	Class4	Class4	Class3	Class1
89	Class1	Class4	Class2	Class1	Class6
90	Class1	Class4	Class4	Class1	Class6

Table S6 The dissolving extent indicator second-level sub-objective pairwise comparison judgment optimality matrix

	Approximate solubility	Cumulative dissolution at each time point	D_{10}、D_{50}、D_{90}	*Span*
Approximate solubility	1(a_{11})	1（a_{12}）	3（a_{13}）	5（a_{14}）
Cumulative dissolution at each time point	1（a_{21}）	1（a_{22}）	3（a_{23}）	5（a_{24}）
D_{10}、D_{50}、D_{90}	1/3（a_{31}）	1/3（a_{32}）	1（a_{33}）	5/3（a_{34}）
Span	1/5（a_{41}）	1/5（a_{42}）	3/5（a_{43}）	1（a_{44}）

Table S7 The taste second-level sub-objective pairwise comparison judgment optimality matrix

	**Turbidity**	D_{90}	**Bitterne-ss**	**Sweetn-ess**	**Saltine-ss**	**Umami**	**Astring-ency**	**Sournes-s**	**Aftertaste-B**	**Aftertaste-A**	**Richnes-s**
Turbidity	1（a_{11}）	1（a_{12}）	1（a_{13}）	1（a_{14}）	5（a_{15}）	3（a_{16}）	5（a_{17}）	3（a_{18}）	7（a_{19}）	7（a_{110}）	7（a_{111}）
D_{90}	1（a_{21}）	1（a_{22}）	1（a_{23}）	1（a_{24}）	5（a_{25}）	3（a_{26}）	5（a_{27}）	3（a_{28}）	7（a_{29}）	7（a_{210}）	7（a_{211}）
Bitterness	1（a_{31}）	1（a_{32}）	1（a_{33}）	1（a_{34}）	5（a_{35}）	3（a_{36}）	5（a_{37}）	3（a_{38}）	7（a_{39}）	7（a_{310}）	7（a_{311}）
Sweetness	1（a_{41}）	1（a_{42}）	1（a_{43}）	1（a_{44}）	5（a_{45}）	3（a_{46}）	5（a_{47}）	3（a_{48}）	7（a_{49}）	7（a_{410}）	7（a_{411}）
Saltiness	1/5（a_{51}）	1/5（a_{52}）	1/5（a_{53}）	1/5（a_{54}）	1（a_{55}）	3/5（a_{56}）	1（a_{57}）	3/5（a_{58}）	7/5（a_{59}）	7/5（a_{510}）	7/5(a_{511})
Umami	1/3(a_{61})	1/3（a_{62}）	1/3（a_{63}）	1/3（a_{64}）	5/3（a_{65}）	1（a_{66}）	5/3（a_{67}）	1（a_{68}）	7/3（a_{69}）	7/3（a_{610}）	7/3（a_{611}）
Astringency	1/5（a_{71}）	1/5（a_{72}）	1/5（a_{73}）	1/5（a_{74}）	1（a_{75}）	3/5（a_{76}）	1（a_{77}）	3/5（a_{78}）	7/5（a_{79}）	7/5（a_{710}）	7/5（a_{711}）
Sourness	1/3（a_{81}）	1/3（a_{82}）	1/3（a_{83}）	1/3（a_{84}）	5/3（a_{85}）	1（a_{86}）	5/3（a_{87}）	1（a_{88}）	7/3（a_{89}）	7/3（a_{810}）	7/3(a_{811})
Aftertaste-B	1/7(a_{91})	1/7（a_{92}）	1/7（a_{93}）	1/7（a_{94}）	5/7（a_{95}）	3/7（a_{96}）	5/7（a_{97}）	3/7（a_{98}）	1（a_{99}）	1（a_{910}）	1(a_{911})
Aftertaste-A	1/7(a_{101})	1/7（a_{102}）	1/7（a_{103}）	1/7（a_{104}）	5/7（a_{105}）	3/7（a_{106}）	5/7（a_{107}）	3/7（a_{108}）	1（a_{109}）	1（a_{1010}）	1（a_{1011}）
Richness	1/7（a_{111}）	1/7（a_{112}）	1/7（a_{113}）	1/7（a_{114}）	5/7（a_{115}）	3/7（a_{116}）	5/7（a_{117}）	3/7（a_{118}）	1（a_{119}）	1（a_{110}）	1（a_{1111}）

Table S8 The evaluation scores for different granules

No.	Cumulative dissolution at each time point (min)						D_{10}	D_{50}	D_{90}	*Span*	Approx-imate solubili-ty	T_{50}	T_e	Turbi-dity	D_{90}	Sourn-ess	Bitter-ness	Astrin-gency	Afterta-ste-B	Aftertas-te-A	Umam-i	Richn-ess	altin-ess	weetn-ess	Compreh-ensive Score
	0.5	1	1.5	2	3	5																			
1	0.0170	0.0183	0.0181	0.0183	0.0188	0.0184	0.0138	0.0128	0.0660	0.0245	0.1250	0.1599	0.1476	0.0607	0.0660	0.0036	0.0533	0.0055	0.0080	0.0075	0.0000	0.0005	0.0119	0.0371	0.9126
2	0.0170	0.0182	0.0190	0.0195	0.0194	0.0192	0.0142	0.0129	0.0571	0.0216	0.1265	0.1491	0.1545	0.0607	0.0571	0.0123	0.0408	0.0020	0.0079	0.0083	0.0057	0.0005	0.0113	0.0574	0.9123
3	0.0133	0.0193	0.0217	0.0219	0.0214	0.0211	0.0144	0.0136	0.0678	0.0233	0.1270	0.1574	0.1395	0.0581	0.0678	0.0134	0.0461	0.0049	0.0078	0.0076	0.0081	0.0005	0.0075	0.0452	0.9287
4	0.0128	0.0162	0.0171	0.0176	0.0174	0.0167	0.0143	0.0142	0.0735	0.0252	0.1306	0.1209	0.1439	0.0607	0.0735	0.0116	0.0381	0.0019	0.0077	0.0082	0.0057	0.0004	0.0111	0.0557	0.8950
5	0.0084	0.0064	0.0048	0.0044	0.0042	0.0017	0.0145	0.0138	0.0288	0.0000	0.1132	0.1388	0.1480	0.0598	0.0288	0.0056	0.0509	0.0049	0.0080	0.0078	0.0014	0.0003	0.0121	0.0509	0.7174
6	0.0182	0.0191	0.0214	0.0196	0.0191	0.0187	0.0144	0.0142	0.0724	0.0240	0.1262	0.1605	0.1533	0.0584	0.0724	0.0076	0.0401	0.0071	0.0062	0.0067	0.0066	0.0027	0.0092	0.0454	0.9432
7	0.0174	0.0163	0.0160	0.0167	0.0173	0.0165	0.0000	0.0030	0.0468	0.0263	0.1260	0.1625	0.1647	0.0607	0.0468	0.0103	0.0465	0.0055	0.0077	0.0078	0.0055	0.0008	0.0083	0.0450	0.8743
8	0.0175	0.0192	0.0205	0.0208	0.0213	0.0214	0.0084	0.0073	0.0441	0.0251	0.0704	0.0786	0.1496	0.0572	0.0441	0.0125	0.0460	0.0048	0.0075	0.0063	0.0079	0.0006	0.0064	0.0314	0.7288
9	0.0152	0.0179	0.0189	0.0192	0.0194	0.0188	0.0095	0.0052	0.0000	0.0229	0.1063	0.1656	0.1626	0.0567	0.0000	0.0150	0.0447	0.0054	0.0077	0.0079	0.0094	0.0008	0.0090	0.0444	0.7826
10	0.0029	0.0027	0.0030	0.0063	0.0114	0.0156	0.0139	0.0130	0.0048	0.0044	0.0846	0.1592	0.1488	0.0546	0.0048	0.0136	0.0454	0.0082	0.0075	0.0071	0.0102	0.0007	0.0055	0.0101	0.7919
11	0.0163	0.0174	0.0183	0.0191	0.0201	0.0205	0.0079	0.0076	0.0523	0.0257	0.1144	0.1461	0.1533	0.0600	0.0523	0.0146	0.0466	0.0054	0.0078	0.0079	0.0089	0.0006	0.0083	0.0463	0.8777
12	0.0184	0.0200	0.0206	0.0207	0.0207	0.0205	0.0141	0.0137	0.0702	0.0244	0.1222	0.1560	0.1521	0.0604	0.0702	0.0139	0.0454	0.0066	0.0071	0.0080	0.0094	0.0008	0.0084	0.0417	0.9454
13	0.0176	0.0174	0.0169	0.0172	0.0172	0.0162	0.0048	0.0038	0.0375	0.0255	0.1248	0.1574	0.1427	0.0607	0.0375	0.0139	0.0408	0.0036	0.0078	0.0077	0.0080	0.0003	0.0078	0.0496	0.8366
14	0.0142	0.0170	0.0185	0.0195	0.0190	0.0185	0.0070	0.0061	0.0473	0.0257	0.1256	0.1541	0.1521	0.0597	0.0473	0.0123	0.0324	0.0012	0.0074	0.0081	0.0058	0.0004	0.0112	0.0548	0.8652
15	0.0172	0.0162	0.0167	0.0178	0.0185	0.0193	0.0146	0.0146	0.0751	0.0257	0.1316	0.1478	0.1529	0.0607	0.0751	0.0126	0.0446	0.0014	0.0079	0.0085	0.0060	0.0007	0.0114	0.0534	0.9503
16	0.0176	0.0189	0.0198	0.0201	0.0207	0.0207	0.0111	0.0109	0.0615	0.0255	0.1099	0.1568	0.1553	0.0605	0.0615	0.0129	0.0462	0.0035	0.0080	0.0082	0.0073	0.0005	0.0100	0.0487	0.9158
17	0.0190	0.0185	0.0185	0.0191	0.0198	0.0200	0.0141	0.0133	0.0555	0.0196	0.1277	0.1226	0.1549	0.0607	0.0555	0.0124	0.0388	0.0009	0.0078	0.0086	0.0068	0.0006	0.0112	0.0544	0.8802
18	0.0126	0.0149	0.0157	0.0171	0.0188	0.0205	0.0146	0.0146	0.0753	0.0259	0.1290	0.1304	0.1622	0.0607	0.0753	0.0122	0.0378	0.0004	0.0076	0.0070	0.0070	0.0008	0.0104	0.0570	0.9278
19	0.0152	0.0178	0.0202	0.0206	0.0207	0.0202	0.0146	0.0145	0.0748	0.0251	0.1288	0.1292	0.1541	0.0607	0.0748	0.0132	0.0431	0.0037	0.0077	0.0075	0.0084	0.0009	0.0093	0.0561	0.9414
20	0.0150	0.0187	0.0195	0.0204	0.0191	0.0194	0.0141	0.0119	0.0566	0.0234	0.1243	0.1562	0.1508	0.0569	0.0566	0.0126	0.0351	0.0045	0.0071	0.0081	0.0078	0.0006	0.0095	0.0383	0.8868
21	0.0145	0.0165	0.0166	0.0175	0.0184	0.0185	0.0140	0.0139	0.0716	0.0247	0.1275	0.1392	0.1533	0.0607	0.0716	0.0120	0.0409	0.0000	0.0081	0.0083	0.0055	0.0003	0.0119	0.0607	0.9261
22	0.0174	0.0170	0.0165	0.0169	0.0174	0.0169	0.0145	0.0143	0.0569	0.0032	0.1275	0.1569	0.1594	0.0605	0.0569	0.0102	0.0452	0.0010	0.0079	0.0081	0.0053	0.0010	0.0113	0.0542	0.8963
23	0.0125	0.0165	0.0184	0.0198	0.0207	0.0207	0.0117	0.0076	0.0509	0.0253	0.0919	0.1511	0.1504	0.0578	0.0509	0.0106	0.0350	0.0056	0.0066	0.0071	0.0073	0.0016	0.0081	0.0382	0.8263

No.	Cumulative dissolution at each time point (min)						D_{10}	D_{50}	D_{90}	*Span*	Approx-imate solubili-ty	T_{50}	T_e	Turbi-dity	D_{90}	Sourn-ess	Bitter-ness	Astrin-gency	Afterta-ste-B	Aftertas-te-A	Umam-i	Richn-ess	altin-ess	weetn-ess	Compreh-ensive Score
	0.5	1	1.5	2	3	5																			
24	0.0126	0.0157	0.0180	0.0198	0.0203	0.0214	0.0133	0.0100	0.0536	0.0246	0.0894	0.1581	0.1517	0.0527	0.0536	0.0129	0.0235	0.0106	0.0037	0.0054	0.0115	0.0027	0.0045	0.0232	0.8128
25	0.0102	0.0085	0.0066	0.0065	0.0080	0.0053	0.0137	0.0094	0.0559	0.0251	0.1107	0.1496	0.1537	0.0599	0.0559	0.0091	0.0548	0.0043	0.0083	0.0083	0.0043	0.0008	0.0099	0.0460	0.8247
26	0.0146	0.0191	0.0201	0.0212	0.0213	0.0203	0.0135	0.0124	0.0632	0.0243	0.0832	0.1566	0.1488	0.0506	0.0632	0.0079	0.0386	0.0092	0.0049	0.0048	0.0069	0.0023	0.0046	0.0204	0.8322
27	0.0073	0.0100	0.0089	0.0098	0.0102	0.0099	0.0140	0.0113	0.0425	0.0218	0.1078	0.1617	0.1517	0.0412	0.0425	0.0142	0.0298	0.0070	0.0052	0.0061	0.0102	0.0008	0.0054	0.0241	0.7534
28	0.0091	0.0131	0.0158	0.0180	0.0195	0.0205	0.0146	0.0144	0.0710	0.0183	0.0959	0.1355	0.1545	0.0554	0.0710	0.0142	0.0210	0.0051	0.0058	0.0060	0.0105	0.0011	0.0058	0.0252	0.8214
29	0.0201	0.0199	0.0197	0.0196	0.0193	0.0186	0.0146	0.0104	0.0564	0.0245	0.1142	0.1477	0.1537	0.0588	0.0564	0.0153	0.0412	0.0057	0.0072	0.0082	0.0112	0.0011	0.0074	0.0335	0.8848
30	0.0056	0.0047	0.0048	0.0049	0.0062	0.0052	0.0143	0.0138	0.0636	0.0203	0.0895	0.1306	0.1468	0.0492	0.0636	0.0147	0.0300	0.0077	0.0052	0.0061	0.0102	0.0018	0.0056	0.0129	0.7174
31	0.0000	0.0000	0.0048	0.0086	0.0131	0.0108	0.0132	0.0077	0.0194	0.0227	0.0739	0.1470	0.1264	0.0174	0.0194	0.0054	0.0495	0.0095	0.0079	0.0063	0.0042	0.0016	0.0055	0.0053	0.5798
32	0.0032	0.0090	0.0133	0.0210	0.0205	0.0212	0.0100	0.0027	0.0282	0.0250	0.0972	0.1434	0.1452	0.0555	0.0282	0.0128	0.0411	0.0121	0.0087	0.0081	0.0092	0.0010	0.0068	0.0371	0.7606
33	0.0020	0.0032	0.0058	0.0083	0.0108	0.0147	0.0101	0.0000	0.0245	0.0253	0.1028	0.0924	0.1102	0.0492	0.0245	0.0077	0.0511	0.0078	0.0074	0.0079	0.0048	0.0007	0.0083	0.0297	0.6091
34	0.0040	0.0134	0.0215	0.0215	0.0170	0.0115	0.0116	0.0034	0.0273	0.0248	0.0896	0.1374	0.0955	0.0432	0.0273	0.0159	0.0339	0.0096	0.0079	0.0080	0.0117	0.0007	0.0062	0.0316	0.6746
35	0.0006	0.0152	0.0183	0.0191	0.0167	0.0159	0.0103	0.0018	0.0273	0.0251	0.0942	0.1281	0.1183	0.0427	0.0273	0.0177	0.0293	0.0116	0.0086	0.0072	0.0125	0.0011	0.0054	0.0298	0.6841
36	0.0118	0.0152	0.0175	0.0160	0.0191	0.0198	0.0026	0.0037	0.0397	0.0258	0.0000	0.0083	0.1667	0.0512	0.0397	0.0140	0.0459	0.0071	0.0072	0.0077	0.0094	0.0004	0.0081	0.0317	0.5687
37	0.0119	0.0107	0.0093	0.0126	0.0089	0.0085	0.0046	0.0050	0.0436	0.0257	0.0284	0.0753	0.1614	0.0543	0.0436	0.0144	0.0414	0.0083	0.0070	0.0074	0.0108	0.0009	0.0074	0.0373	0.6387
38	0.0107	0.0140	0.0154	0.0179	0.0195	0.0202	0.0029	0.0041	0.0409	0.0258	0.0175	0.0000	0.1529	0.0546	0.0409	0.0142	0.0512	0.0076	0.0078	0.0075	0.0086	0.0005	0.0085	0.0297	0.5730
39	0.0139	0.0152	0.0153	0.0153	0.0160	0.0156	0.0042	0.0046	0.0439	0.0258	0.0127	0.0467	0.1541	0.0547	0.0439	0.0140	0.0441	0.0093	0.0079	0.0077	0.0100	0.0008	0.0070	0.0355	0.6181
40	0.0134	0.0148	0.0169	0.0186	0.0194	0.0196	0.0058	0.0055	0.0429	0.0255	0.0169	0.0508	0.1427	0.0497	0.0429	0.0176	0.0406	0.0100	0.0081	0.0077	0.0117	0.0004	0.0063	0.0380	0.6260
41	0.0116	0.0144	0.0153	0.0129	0.0101	0.0177	0.0056	0.0054	0.0428	0.0256	0.0132	0.0629	0.1598	0.0446	0.0428	0.0175	0.0459	0.0086	0.0077	0.0076	0.0116	0.0002	0.0068	0.0401	0.6306
42	0.0013	0.0130	0.0154	0.0197	0.0216	0.0180	0.0051	0.0049	0.0402	0.0255	0.0049	0.1472	0.1529	0.0000	0.0402	0.0143	0.0439	0.0076	0.0074	0.0070	0.0120	0.0017	0.0063	0.0323	0.6422
43	0.0219	0.0212	0.0219	0.0209	0.0203	0.0199	0.0032	0.0044	0.0441	0.0259	0.0203	0.0384	0.1517	0.0566	0.0441	0.0137	0.0507	0.0072	0.0072	0.0081	0.0088	0.0003	0.0080	0.0342	0.6530
44	0.0053	0.0086	0.0106	0.0122	0.0155	0.0161	0.0140	0.0110	0.0523	0.0236	0.0869	0.1578	0.1537	0.0464	0.0523	0.0146	0.0414	0.0099	0.0080	0.0086	0.0098	0.0008	0.0075	0.0397	0.8068
45	0.0046	0.0074	0.0098	0.0141	0.0188	0.0213	0.0135	0.0084	0.0321	0.0233	0.0910	0.1569	0.1521	0.0441	0.0321	0.0157	0.0419	0.0066	0.0071	0.0083	0.0110	0.0009	0.0081	0.0377	0.7668
46	0.0110	0.0153	0.0196	0.0210	0.0215	0.0219	0.0143	0.0137	0.0605	0.0190	0.1027	0.1653	0.1525	0.0506	0.0605	0.0148	0.0366	0.0069	0.0072	0.0083	0.0101	0.0008	0.0087	0.0430	0.8857
47	0.0002	0.0013	0.0017	0.0047	0.0096	0.0155	0.0141	0.0132	0.0617	0.0222	0.0827	0.1112	0.1224	0.0449	0.0617	0.0120	0.0482	0.0076	0.0073	0.0071	0.0080	0.0009	0.0082	0.0159	0.6824

No.	Cumulative dissolution at each time point (min)						D_{10}	D_{50}	D_{90}	*Span*	Approx-imate solubili-ty	T_{50}	T_e	Turbi-dity	D_{90}	Sourn-ess	Bitter-ness	Astrin-gency	Afterta-ste-B	Aftertas-te-A	Umam-i	Richn-ess	altin-ess	weetn-ess	Compreh-ensive Score
	0.5	1	1.5	2	3	5																			
48	0.0072	0.0095	0.0109	0.0149	0.0181	0.0207	0.0140	0.0125	0.0454	0.0196	0.0998	0.1052	0.1460	0.0509	0.0454	0.0146	0.0389	0.0067	0.0071	0.0080	0.0109	0.0012	0.0082	0.0404	0.7563
49	0.0018	0.0049	0.0064	0.0127	0.0164	0.0153	0.0139	0.0121	0.0491	0.0215	0.0951	0.1640	0.1427	0.0508	0.0491	0.0145	0.0493	0.0079	0.0074	0.0087	0.0103	0.0006	0.0078	0.0346	0.7971
50	0.0057	0.0072	0.0089	0.0127	0.0166	0.0174	0.0121	0.0088	0.0339	0.0233	0.0504	0.1296	0.1386	0.0447	0.0339	0.0141	0.0277	0.0112	0.0084	0.0071	0.0111	0.0013	0.0055	0.0272	0.6575
51	0.0075	0.0180	0.0185	0.0174	0.0188	0.0157	0.0141	0.0137	0.0687	0.0234	0.0953	0.1655	0.1053	0.0503	0.0687	0.0117	0.0353	0.0116	0.0049	0.0036	0.0114	0.0042	0.0041	0.0167	0.8043
52	0.0216	0.0219	0.0219	0.0216	0.0219	0.0211	0.0141	0.0141	0.0729	0.0251	0.0991	0.1658	0.1366	0.0548	0.0729	0.0117	0.0290	0.0104	0.0040	0.0026	0.0119	0.0046	0.0039	0.0168	0.8805
53	0.0129	0.0153	0.0149	0.0140	0.0136	0.0124	0.0141	0.0136	0.0681	0.0236	0.1103	0.1648	0.1012	0.0562	0.0681	0.0125	0.0322	0.0103	0.0047	0.0038	0.0118	0.0036	0.0041	0.0146	0.8006
54	0.0159	0.0149	0.0145	0.0142	0.0136	0.0115	0.0142	0.0141	0.0724	0.0247	0.1022	0.1647	0.1069	0.0553	0.0724	0.0125	0.0333	0.0110	0.0045	0.0036	0.0119	0.0037	0.0037	0.0150	0.8104
55	0.0084	0.0170	0.0194	0.0202	0.0215	0.0214	0.0144	0.0143	0.0739	0.0250	0.1023	0.1644	0.1143	0.0534	0.0739	0.0119	0.0337	0.0108	0.0046	0.0034	0.0119	0.0043	0.0039	0.0162	0.8448
56	0.0028	0.0146	0.0171	0.0171	0.0173	0.0177	0.0143	0.0142	0.0735	0.0250	0.0971	0.1605	0.0899	0.0492	0.0735	0.0118	0.0303	0.0106	0.0048	0.0040	0.0124	0.0039	0.0045	0.0151	0.7813
57	0.0082	0.0182	0.0204	0.0207	0.0208	0.0218	0.0145	0.0145	0.0729	0.0209	0.0979	0.1640	0.1203	0.0534	0.0729	0.0130	0.0310	0.0110	0.0046	0.0037	0.0124	0.0039	0.0040	0.0186	0.8437
58	0.0051	0.0123	0.0141	0.0136	0.0133	0.0120	0.0143	0.0140	0.0702	0.0230	0.0920	0.1630	0.1130	0.0496	0.0702	0.0121	0.0329	0.0108	0.0047	0.0042	0.0113	0.0035	0.0043	0.0173	0.7809
59	0.0088	0.0157	0.0208	0.0212	0.0212	0.0219	0.0143	0.0142	0.0727	0.0242	0.0987	0.1658	0.1021	0.0549	0.0727	0.0119	0.0350	0.0111	0.0048	0.0038	0.0116	0.0041	0.0042	0.0167	0.8323
60	0.0142	0.0191	0.0202	0.0205	0.0208	0.0204	0.0141	0.0115	0.0431	0.0216	0.0874	0.1667	0.1118	0.0499	0.0431	0.0127	0.0366	0.0112	0.0051	0.0040	0.0120	0.0037	0.0042	0.0175	0.7714
61	0.0093	0.0108	0.0105	0.0149	0.0116	0.0128	0.0146	0.0141	0.0538	0.0074	0.1083	0.1363	0.1549	0.0522	0.0538	0.0173	0.0175	0.0086	0.0036	0.0055	0.0126	0.0018	0.0041	0.0213	0.7574
62	0.0047	0.0079	0.0087	0.0102	0.0141	0.0165	0.0109	0.0089	0.0516	0.0251	0.0685	0.0898	0.1077	0.0340	0.0516	0.0193	0.0306	0.0066	0.0056	0.0014	0.0146	0.0017	0.0001	0.0262	0.6161
63	0.0096	0.0150	0.0161	0.0169	0.0175	0.0174	0.0140	0.0121	0.0553	0.0228	0.1254	0.1559	0.1504	0.0583	0.0553	0.0171	0.0000	0.0110	0.0000	0.0031	0.0132	0.0028	0.0034	0.0278	0.8204
64	0.0027	0.0020	0.0000	0.0000	0.0000	0.0000	0.0114	0.0093	0.0491	0.0246	0.1190	0.1585	0.1370	0.0593	0.0491	0.0166	0.0279	0.0058	0.0050	0.0010	0.0166	0.0054	0.0024	0.0313	0.7340
65	0.0130	0.0164	0.0185	0.0206	0.0181	0.0199	0.0106	0.0077	0.0383	0.0244	0.1003	0.1146	0.1391	0.0579	0.0383	0.0167	0.0217	0.0026	0.0063	0.0054	0.0125	0.0010	0.0065	0.0305	0.7407
66	0.0042	0.0098	0.0135	0.0176	0.0184	0.0197	0.0083	0.0035	0.0311	0.0251	0.1146	0.1591	0.1474	0.0583	0.0311	0.0170	0.0446	0.0074	0.0073	0.0080	0.0111	0.0005	0.0074	0.0326	0.7974
67	0.0084	0.0103	0.0101	0.0116	0.0121	0.0136	0.0130	0.0112	0.0561	0.0242	0.1124	0.1588	0.1447	0.0550	0.0561	0.0190	0.0364	0.0081	0.0066	0.0058	0.0133	0.0005	0.0040	0.0269	0.8181
68	0.0116	0.0153	0.0177	0.0191	0.0191	0.0191	0.0142	0.0121	0.0567	0.0231	0.0763	0.1550	0.1244	0.0517	0.0567	0.0156	0.0263	0.0059	0.0028	0.0022	0.0111	0.0021	0.0000	0.0144	0.7525
69	0.0118	0.0132	0.0137	0.0146	0.0154	0.0149	0.0142	0.0140	0.0698	0.0228	0.1185	0.1559	0.1384	0.0582	0.0698	0.0150	0.0510	0.0101	0.0067	0.0069	0.0096	0.0013	0.0048	0.0101	0.8606
70	0.0134	0.0146	0.0150	0.0160	0.0166	0.0157	0.0143	0.0134	0.0576	0.0199	0.1273	0.1579	0.1545	0.0578	0.0576	0.0150	0.0255	0.0117	0.0035	0.0043	0.0117	0.0025	0.0042	0.0249	0.8549
71	0.0077	0.0103	0.0107	0.0129	0.0141	0.0150	0.0119	0.0097	0.0460	0.0240	0.1003	0.1658	0.1409	0.0589	0.0460	0.0146	0.0351	0.0053	0.0068	0.0068	0.0097	0.0006	0.0068	0.0335	0.7934

No.	Cumulative dissolution at each time point (min)						D_{10}	D_{50}	D_{90}	*Span*	Approx-imate solubili-ty	T_{50}	T_e	Turbi-dity	D_{90}	Sourn-ess	Bitter-ness	Astrin-gency	Afterta-ste-B	Aftertas-te-A	Umam-i	Richn-ess	altin-ess	weetn-ess	Compreh-ensive Score
	0.5	1	1.5	2	3	5																			
72	0.0052	0.0080	0.0065	0.0085	0.0100	0.0078	0.0119	0.0088	0.0410	0.0240	0.0810	0.1559	0.1403	0.0576	0.0410	0.0000	0.0607	0.0103	0.0075	0.0045	0.0003	0.0012	0.0059	0.0118	0.7097
73	0.0083	0.0110	0.0136	0.0159	0.0183	0.0195	0.0127	0.0102	0.0542	0.0246	0.0928	0.1544	0.1232	0.0553	0.0542	0.0162	0.0167	0.0087	0.0032	0.0053	0.0107	0.0011	0.0056	0.0228	0.7586
74	0.0128	0.0147	0.0155	0.0161	0.0175	0.0190	0.0088	0.0080	0.0523	0.0255	0.1150	0.1506	0.1409	0.0601	0.0523	0.0160	0.0495	0.0054	0.0081	0.0085	0.0103	0.0006	0.0077	0.0318	0.8472
75	0.0076	0.0091	0.0090	0.0111	0.0135	0.0159	0.0146	0.0144	0.0738	0.0243	0.1109	0.1608	0.1431	0.0421	0.0738	0.0160	0.0411	0.0056	0.0058	0.0000	0.0130	0.0029	0.0013	0.0204	0.8302
76	0.0062	0.0085	0.0108	0.0132	0.0161	0.0203	0.0143	0.0141	0.0709	0.0234	0.1045	0.1573	0.1368	0.0420	0.0709	0.0157	0.0283	0.0119	0.0040	0.0037	0.0129	0.0030	0.0043	0.0277	0.8207
77	0.0166	0.0174	0.0168	0.0185	0.0207	0.0212	0.0136	0.0114	0.0597	0.0245	0.1229	0.1585	0.0854	0.0517	0.0597	0.0144	0.0595	0.0058	0.0071	0.0017	0.0098	0.0014	0.0007	0.0000	0.7991
78	0.0097	0.0107	0.0134	0.0159	0.0186	0.0194	0.0143	0.0134	0.0591	0.0202	0.0690	0.0514	0.0000	0.0248	0.0591	0.0164	0.0482	0.0103	0.0072	0.0039	0.0159	0.0082	0.0046	0.0271	0.5406
79	0.0103	0.0093	0.0085	0.0094	0.0102	0.0095	0.0143	0.0136	0.0587	0.0187	0.0999	0.1295	0.1220	0.0521	0.0587	0.0202	0.0330	0.0073	0.0048	0.0041	0.0142	0.0017	0.0006	0.0233	0.7340
80	0.0150	0.0150	0.0155	0.0163	0.0165	0.0170	0.0143	0.0140	0.0556	0.0118	0.1280	0.1646	0.1573	0.0607	0.0556	0.0124	0.0534	0.0067	0.0078	0.0080	0.0075	0.0006	0.0082	0.0342	0.8961
81	0.0099	0.0111	0.0118	0.0133	0.0155	0.0158	0.0095	0.0086	0.0517	0.0253	0.0694	0.0722	0.1183	0.0500	0.0517	0.0154	0.0469	0.0075	0.0071	0.0070	0.0089	0.0000	0.0056	0.0245	0.6570
82	0.0102	0.0109	0.0108	0.0109	0.0121	0.0120	0.0132	0.0113	0.0597	0.0247	0.0944	0.1199	0.1407	0.0450	0.0597	0.0145	0.0418	0.0067	0.0071	0.0072	0.0088	0.0014	0.0071	0.0421	0.7721
83	0.0105	0.0110	0.0110	0.0118	0.0134	0.0131	0.0145	0.0142	0.0716	0.0229	0.1275	0.1488	0.1330	0.0594	0.0716	0.0161	0.0348	0.0083	0.0060	0.0067	0.0155	0.0050	0.0045	0.0268	0.8582
84	0.0062	0.0062	0.0058	0.0063	0.0074	0.0076	0.0144	0.0142	0.0722	0.0236	0.0871	0.1626	0.0370	0.0479	0.0722	0.0190	0.0479	0.0085	0.0073	0.0026	0.0202	0.0087	0.0034	0.0248	0.7132
85	0.0076	0.0063	0.0050	0.0056	0.0064	0.0057	0.0136	0.0118	0.0574	0.0237	0.0770	0.1620	0.1429	0.0539	0.0574	0.0177	0.0490	0.0071	0.0076	0.0074	0.0128	0.0014	0.0068	0.0385	0.7846
86	0.0082	0.0074	0.0068	0.0074	0.0084	0.0077	0.0139	0.0129	0.0607	0.0226	0.1004	0.1547	0.1482	0.0523	0.0607	0.0139	0.0345	0.0089	0.0054	0.0055	0.0107	0.0016	0.0053	0.0222	0.7805
87	0.0059	0.0065	0.0069	0.0092	0.0142	0.0169	0.0138	0.0135	0.0675	0.0236	0.1143	0.1392	0.1439	0.0581	0.0675	0.0156	0.0218	0.0076	0.0045	0.0049	0.0117	0.0019	0.0051	0.0271	0.8011
88	0.0111	0.0138	0.0141	0.0163	0.0206	0.0213	0.0137	0.0125	0.0611	0.0236	0.0988	0.1597	0.1527	0.0379	0.0611	0.0159	0.0316	0.0081	0.0058	0.0072	0.0119	0.0013	0.0061	0.0210	0.8272
89	0.0143	0.0160	0.0164	0.0172	0.0180	0.0186	0.0145	0.0125	0.0556	0.0221	0.1248	0.1559	0.1521	0.0565	0.0556	0.0153	0.0370	0.0074	0.0052	0.0010	0.0130	0.0043	0.0032	0.0240	0.8602
90	0.0117	0.0123	0.0115	0.0120	0.0134	0.0145	0.0143	0.0132	0.0569	0.0204	0.1010	0.1649	0.1496	0.0575	0.0569	0.0188	0.0395	0.0084	0.0069	0.0080	0.0130	0.0007	0.0048	0.0255	0.8359
